# Lethal and behavioral effects of synthetic and organic insecticides on *Spodoptera exigua* and its predator *Podisus maculiventris*

**DOI:** 10.1371/journal.pone.0206789

**Published:** 2018-11-08

**Authors:** Ancidériton Antonio de Castro, Jesusa Crisostomo Legaspi, Wagner de Souza Tavares, Robert L. Meagher, Neil Miller, Lambert Kanga, Muhammad Haseeb, José Eduardo Serrão, Carlos Frederico Wilcken, José Cola Zanuncio

**Affiliations:** 1 Departamento de Entomologia/BIOAGRO, Universidade Federal de Viçosa, Viçosa, Minas Gerais, Brasil; 2 United States Department of Agriculture—Agricultural Research Service, CMAVE/Florida A&M University—Center for Biological Control, Tallahassee, Florida, United States of America; 3 Departamento de Fitotecnia/BIOAGRO, Universidade Federal de Viçosa, Viçosa, Minas Gerais, Brasil; 4 United States Department of Agriculture—Agricultural Research Service, Center for Medical, Agricultural and Veterinary Entomology, Gainesville, Florida, United States of America; 5 Center for Biological Control, College of Agriculture and Food Sciences, Florida A&M University, Tallahassee, Florida, United States of America; 6 Departamento de Biologia Geral, Universidade Federal de Viçosa, Viçosa, Minas Gerais, Brasil; 7 Departamento de Proteção Vegetal, Faculdade de Ciências Agronômicas, Universidade Estadual Paulista “Júlio de Mesquita Filho”, Botucatu, São Paulo, Brasil; Chinese Academy of Agricultural Sciences, CHINA

## Abstract

**Background:**

The beet armyworm, *Spodoptera exigua* (Lepidoptera: Noctuidae), is a key insect pest of edible vegetables around the world and it is resistant to insecticide of different classes. Insecticides that are effective to this pest and selective to predator stinkbugs are required for the integrated management of *S*. *exigua*.

**Methods:**

The toxicity of four commercial insecticide formulations azadirachtin + pyrethrin, spinosad, pyrethrin and chlorantraniliprole was tested on the target pest and their side effect were evaluated on the spined soldier bug, *Podisus maculiventris* (Heteroptera: Pentatomidae) through different bioassays.

**Results:**

Spinosad and chlorantraniliprole were more toxic to *S*. *exigua* than to the predator *P*. *maculiventris* but opposite results were obtained for pyrethrin and azadirachtin + pyrethrin in contact toxicity bioassay. Chlorantraniliprole was the most toxic to *S*. *exigua* in oral toxicity bioassay, followed by spinosad, pyrethrin and azadirachtin + pyrethrin. Spinosad in oral toxicity bioassay was the most toxic to *P*. *maculiventris*, followed by pyrethrin, azadirachtin + pyrethrin and chlorantraniliprole. Spinosad caused irritability to the predator while pyrethrin to the pest. The insecticide repellency was not observed over the tested insect species. The synthetic insecticide chlorantraniliprole was less toxic than the natural pyrethrin, azadirachtin + pyrethrin and spinosad to the predator.

**Conclusions:**

This work provides useful information on the combination of commercial insecticides with the predator *P*. *maculiventris* to controlling *S*. *exigua* in integrated pest management (IPM) programs.

## Introduction

The beet armyworm, *Spodoptera exigua* (Hübner) (Lepidoptera: Noctuidae), native to Southeast Asia, is an important insect pest of edible vegetables in several regions of the world [1,2]. The species damages (e.g. defoliation) numerous cultivated crops including corn, cotton, onion, peanut, potato, soybean, and tomato [3,4]. Crop losses are important especially in tomato production due to *S*. *exigua* attacks with an economic injury level of one caterpillar per 20 plants based on an early season infestation [5]. This pest is mainly controlled by insecticides, but resistance phenomena can reduce the efficiency of the active ingredients [6–8]. In this case, the development of alternative control methods for the integrated management of *S*. *exigua* is needed [9,10]. Selective insecticides to natural enemies is important because these organisms are promising for the management of this pest in both field and greenhouse cropping systems [11,12].

The spined soldier bug, *Podisus maculiventris* (Say) (Heteroptera: Pentatomidae), is one of the most common generalist predators of *S*. *exigua* [11]. This natural enemy has a great predation capacity even at high pest densities over increasing temperatures [10]. Side effect evaluation of pesticides when *P*. *maculiventris* is used in the integrated *S*. *exigua* management context is important. Insecticides are the most toxic pesticide class to insect natural enemies, followed by herbicides, acaricides and fungicides, respectively. Among insecticide classes, a trend of increasing toxicity to natural enemies is present from the early inorganics to the synthetic pyrethroids [13]. More recent botanicals, microbials and insect growth regulators seem to have lower toxicity and are more selective. However, the susceptibility trends among pests and natural enemies are variable [14]. Studies on side-effects of pesticides on natural enemies are important and they include lethal and sublethal assays assessing the acute toxicity and their effect on the ephysiology and the behavior of these insects, respectively [15]. Fourth instar *P*. *maculiventris* was affected negatively by teflubenzuron (insect growth regulator; acylureas); both 4^th^ instar nymphs and females died when they were exposed to methomyl (carbamate insecticide); a marked decline in egg hatch was observed when the predators were exposed to teflubenzuron [16]. *Podisus maculiventris* was affected negatively by cyfluthrin (pyrethroid insecticide) and oxamyl (carbamate insecticide) in residual and feeding tests, respectively; feeding on indoxacarb-treated food (oxadiazine insecticide) caused mortality for both nymphs and adults of this predator [17]. The impact of the insecticides tested on non-target species needs further studies.

Pesticides used in organic farming are used in integrated management programs of *S*. *exigua* showing the importance of defining the susceptibility of natural enemies and pests to these products. The objective of this study was to evaluate the toxicity and the behavioral response of *S*. *exigua* and the predator *P*. *maculiventris* to insecticides. The tested insecticides were those used in organic farming azadirachtin + pyrethrin, pyrethrin, spinosad and the synthetic formulation, chlorantraniliprole. This study can improve the integrated use of insecticides, with low toxicity to the main predator, *P*. *maculiventris*, of *S*. *exigua*.

## Material and methods

### Insect rearing

*Spodoptera exigua* colony was obtained from the United States Department of Agriculture (USDA), Agricultural Research Service (ARS), Center for Medical, Agriculture and Veterinary (CMAVE), in Gainesville, FL, USA. *Spodoptera exigua* larvae were reared on an artificial diet based on beans [10]. The predator *P*. *maculiventris* was obtained from a laboratory colony of the same institution in Tallahassee, FL, USA. This natural enemy was fed with the yellow mealworm larvae, *Tenebrio molitor* L. (Coleoptera: Tenebrionidae) and received water. *Podisus maculiventris* nymphs and *S*. *exigua* larvae were observed daily to obtain 3^rd^ instar individuals of these species for the bioassays. All insects were kept in a room equipped with air conditioner and humidifier at 25 ± 2°C, 70 ± 5% RH and 12L:12D photoperiod. Yellow mealworm larvae and adults were reared in plastic tray with a mixture of 95% wheat flour and 5% yeast, in addition to carrots and sweet potatoes as a food, supplied once a week [18].

### Insecticides

Four insecticides were tested (three used in organic farm and one synthetic) for *S*. *exigua* and *P*. *maculiventris*. The tested active ingredients and their commercial formulations were: pyrethrin (PyGanic Crop Protection EC 5.0 II; 50 g a.i. L^–1^; McLaughlin Gormley King Co.; Minneapolis, MN, USA), spinosad (Entrust; 240 g a.i. L^–1^; Dow AgroSciences; Indianapolis, IN, USA), azadirachtin + pyrethrin (Azera; 12 g a.i. azadirachtin L^–1^ and 13.2 g a.i. pyrethrin L^–1^; McLaughlin Gormley King Co.; Minneapolis, MN, USA) and chlorantraniliprole (Coragen; 200 g a.i. L^–1^; DuPont; Wilmington, DE, USA) (Table 1).

**Table 1:** Active ingredient (a.i.), formulation concentration (FC), recommended field concentration (RFC), authorized in organic farming (yes or not, AOF), company, and country of brand name (BN) products.

BN	Coragen	Entrust	PyGanic Crop Protection EC 5.0 II	Azera
a.i.	Chlor.	Spinosad	Pyr.	Azad.+Pyr.
FC(g a.i. L^–1^)	200	240	50	12 Azad.+13.2 Pyr.
RFC(μg a.i. mL^–1^)	500	1,486.1	87.9	99.80 Azad.+109.81 Pyr.
AOF	not	yes		
Company	DuPont	Dow AgroSciences	McLaughlin Gormley King Co.	
Country	USA			

Azadirachtin + pyrethrin = Azad. + Pyr., pyrethrin = Pyr. and chlorantraniliprole = Chlor.

PyGanic Crop Protection EC 5.0 II has potent insecticidal activity acting on the insect nervous systems and it is allowed to be used in a range of crops against several pest species [19]. Entrust is a highly active microbial insecticide with neural mechanism, by both contact and ingestion, in numerous insect species, including coleopteran and lepidopteran pests in various crops [20,21]. Azera acts as a sodium channel inhibitor and disrupts the insect nervous system as a growth regulator in a range of insects attacking several crops [22]. PyGanic Crop Protection EC 5.0 II, Entrust and Azera are compliant with the National Organic Program (NOP) of the United States Department of Agriculture (USDA) and Agricultural Marketing Service (AMS) requirements for an organic farm and approved by the Organic Materials Review Institute (OMRI). Coragen, an insecticide not listed by the OMRI, has wide activity against coleopteran, dipteran, hemipteran, isopteran, and lepidopteran pests and its use is allowed to control several crop pests [21,23,24], including *S*. *exigua* [25]. All the tested products in the present study were authorized for the control of *S*. *exigua* in USA. Our study included three different kinds of bioassays (contact toxicity on glass, oral toxicity and behavioral response) in pesticide lethal and subletal evaluations.

### Contact toxicity on glass

The bioassay evaluated the effect of direct contact of insecticides to insect body on *S*. *exigua* and *P*. *maculiventris* mortality. This bioassay was conducted in 20 mL glass vials treated with a 0.5 mL solution of each insecticide according to the treatment. The vials were stirred until the water evaporated and the insecticides covered the inner vial surface [26]. The control had vials treated with distilled water. The bioassay was designed according a completely randomized design between five to eight concentrations and six replications per insecticide. Dilution ratios of insecticide solutions between 1:1 to 1:10^5^ were tested. Each insecticide was diluted in distilled water to obtain the desired concentration.

Per each replication, three larvae of 3^rd^ instar *S*. *exigua* or three nymphs of 3^rd^ instar *P*. *maculiventris* were individualized in the glass vial treated with each treatment and were kept at 25 ± 2°C, 70 ± 5% RH and 12L:12D photoperiod. *Spodoptera exigua* and *P*. *maculiventris* mortality was determined 24 h after exposure; larvae (pest) and nymphs (predator) unable to walk up to 10 mm when released were considered dead [27].

### Oral toxicity

The bioassay evaluated the effect of insecticide ingestion through artificial diet and drinking water on the mortality of *S*. *exigua* and *P*. *maculiventris*, respectively. The dilution of the insecticides from 1:1 to 1:10^5^ was prepared in distilled water to obtain the desired concentrations. The bioassay was set up with a completely randomized experimental design with five to eight insecticide concentrations with six replications. The procedure used to make serial dilutions was the same for both the pest and its predator. The bioassay was carried out simultaneously under the same conditions. Insecticide toxicity was evaluated on 3^rd^ instar *S*. *exigua* larvae and 3^rd^ instar *P*. *maculiventris* nymphs. The insecticide was incorpored into the diet used to feed the caterpillar. After the preparation of the diet, 0.5 mL of each diluted insecticide solution (or distilled water) was mixed with 1.5 g of artificial diet in a plastic cup (1 oz.). On the other hand, predatory nymphs were exposed to the concentrations of each insecticide solution by ingestion in treated distilled water. The insecticides were supplied to the predators in 0.5 mL glass cylindrical tubes with cotton soaked at the bottom of the tubes, inserted in the cup lid.

Five replications were used per insecticide solution, in addition to the control treatment (i.e. distilled water). Seven concentrations per insecticide with serial dilutions were prepared with distilled water. Five 3^rd^ instar *S*. *exigua* larvae or one 3^rd^ instar *P*. *maculiventris* nymph were placed per cup with 10 cups per insecticide concentration. Three hundred and fifty pest larvae and 210 predator nymphs were used per treatment. Nymphs of *P*. *maculiventris* were starved within 24 h prior to initiation and during the experiment to stimulate the drinking behavior of this predator. The cups were covered with paper lids and kept at 25 ± 2°C, 70 ± 5% RH and 12L:12D photoperiod. The insect mortality was evaluated 24 h after larvae and nymphs were placed in the cups. The *S*. *exigua* larvae [28] and *P*. *maculiventris* nymphs [21] were considered dead when they responded neither with head movements nor with peristaltic contractions, after being touched with a camel-hair brush [28].

### Behavioral response

The bioassays evaluated the effect of direct insecticide contact to insect body on the *S*. *exigua* and *P*. *maculiventris* behavior. Two behavioral locomotion bioassays were performed with 3^rd^ instar *S*. *exigua* larvae and 3^rd^ instar *P*. *maculiventris* nymphs in Petri dishes (9 cm diameter × 2 cm height) with half of the base treated and the other half untreated with insecticide [21,29,30]. An untreated filter paper disk (control) (Whatman No. 1; 9 cm diameter; Sigma-Aldrich; St. Louis, MO, USA) was placed on the base of the dish and the half of another disk treated with insecticide solution fixed with water-based synthetic white glue Maxi Cola (Frama; Caxias do Sul, Rio Grande do Sul, Brazil) on the control disk. The glue did not affect the behavior of insects, as observed in previous trials. Filter papers were treated with the recommended field concentration of each insecticide (Table 1). The insecticide concentrations used were those for the field, because no mortality was observed during the 10 minutes of exposure in the treatments, including the control. The insects of each species were placed on the dish with the inner side covered with Teflon PTFE (DuPont; Wilmington, DE, USA) to avoid their escape. The filter paper disk was dipped for five seconds into 1 mL solution corresponding to each insecticide concentration recommended for the field. The experiments had a completely randomized design with 20 insects (replications) per insecticide solution and insect species (including the control) in half-treated dish bioassays. For each replication, the filter paper was replaced and the side on which the insect was released on the dish was randomly established by test.

The insect movement per dish was recorded for 10 minutes with a Sony Handycam DCR-SR68 camera on a tripod at a height of approximately 60 cm above the dish. Digital recordings were transferred to computer and analyzed with EthoVision XT software (v.7.1 Noldus; Wageningen, The Netherlands). The images of the dish were divided into two symmetrical zones (one treated and one not). The distance walked (cm) in treated or untreated zones, time spent in each zone and walking speed (cm s^–1^) of the insect on each half of the dish were recorded. Insects that spent less than one second on the insecticide-treated half of the dish were considered repelled and those that stayed less than 50% of the time in the treated half were considered irritated [31].

### Statistical analysis

Homogeneity of variance and normality of errors were verified and no data transformation was required (PROC UNIVARIATE; GPLOT PROC). The mortality data for both contact toxicity on glass and oral toxicity bioassays were subjected to Probit analysis [32]. The concentration-mortality relationships were considered true when there was no significant deviation (*P* > 0.05). The mortality percentage was also corrected for the control using Abbott formula: [(the percent living in the check–the percent living in the treated plat) ÷ the percent living in the check] × 100 [33]. The selectivity and toxicity rates were calculated [34]. The relative toxicity (RT) was calculated by the higher lethal concentration, 50% (LC_50_) value of insecticide (least toxic)/lower LC_50_ value (most toxic) of the other insecticides [35]. To measure the insecticide selectivity for *P*. *maculiventris*, we calculated the differential selectivity with 95% confidence intervals based on the LC_50_ values of the insecticides for *S*. *exigua* and *P*. *maculiventris*. The differential selectivity was calculated by LC_50_ value of each insecticide (predator)/LC_50_ value (pest) of the same insecticide. Differences between insecticides were considered significant if the 95% confidence level of LC_50_ did not overlap [36]. Differences in time spent on each half of the semi-treated dishes (insecticide avoidance) were tested using paired Student’s *t* test (*P* < 0.05) per insecticide and insect species. Analyses were processed using the software SAS, version 9.2 [37].

### Statement

No specific permits are required to rear *S*. *exigua*, *P*. *maculiventris* and *T*. *molitor* in the USA. The laboratory studies did not involve endangered or protected species.

## Results

### Contact toxicity on glass

The chlorantraniliprole was the most toxic to *S*. *exigua*, followed by the spinosad, pyrethrin and azadirachtin + pyrethrin, with relative toxicity of 1.00, 10.99, 16.75 and 28.19, respectively. No *P*. *maculiventris* mortality was observed with chlorantraniliprole; therefore, the LC_50_ of this treatment was not calculated. Pyrethrin and azadirachtin + pyrethrin were more toxic than the spinosad to *P*. *maculiventris* by contact with treated glass vials. Azadirachtin + pyrethrin, spinosad and, mainly, chlorantraniliprole were more toxic to the pest than to the predator while pyrethrin toxicity to both the pest and predator was similar (Table 2).

**Table 2:** Relative toxicity (RT) of insecticides to the 3^rd^ instar beet armyworm, *Spodoptera exigua* and RT and differential selectivity (DS) (related to the toxicity data of the beet armyworm) to the 3^rd^ instar spined soldier bug, *Podisus maculiventris* in contact toxicity on glass bioassay at 95% confidence interval.

Species	Insecticide	No.	Slope±SE	LC_50_(95% FL)	RT(95% CI)	DS(95% CI)	χ^2^(*df*)	*P*
*Spodoptera exigua*	Chlor.	288	0.97±0.10	0.35(0.24–0.54)	1.00(0.57–1.76)	-	10.00(30)	0.99
	Spinosad	252	1.63±0.17	3.93(2.98–5.13)	10.99(6.81–17.73)	-	7.95(26)	0.99
	Pyr.	252	1.45±0.16	5.99(4.48–8.08)	16.75(10.25–27.93)	-	8.04(26)	0.99
	Azad.+Pyr.	252	1.66±0.17	10.07(7.71–13.12)	28.19(17.52–45.35)	-	8.56(26)	0.99
*Podisus maculiventris*	Chlor.	300	-	-	-	-	-	-
	Pyr.	234	1.47±0.19	8.51(6.09–11.34)	1.00(0.65–1.53)	1.42(0.94–2.16)	4.63(16)	0.99
	Azad.+Pyr.	288	1.26±0.14	40.85(29.73–58.61)	4.80(3.07–7.51)	4.06(2.66–6.18)	9.16(30)	1.00
	Spinosad	180	2.22±0.29	66.62(52.92–85.35)	7.83(5.36–11.44)	16.96(11.93–24.12)	6.72(18)	0.99

RT, relative toxicity. DS, differential selectivity. FL, fiducial limit. *df*, degree of freedom. CI, confidence interval. No. = number of insects. LC_50_ = LC_50_ (95% FL) μg a.i./vial. Azadirachtin + pyrethrin = Azad. + Pyr., pyrethrin = Pyr. and chlorantraniliprole = Chlor. RT = >LC_50_ (least toxic)/< LC_50_ (most toxic). DS = LC_50_ value of each insecticide (predator)/LC_50_ value (pest) of the same insecticide.

### Oral toxicity

The chlorantraniliprole was the most toxic to *S*. *exigua* in oral toxicity bioassay, followed by the spinosad, pyrethrin and azadirachtin + pyrethrin, with relative toxicity of 1.00, 5.24, 11.98 and 15.13, respectively (Table 3).

**Table 3:** Relative toxicity (RT) of insecticides to the 3^rd^ instar beet armyworm, *Spodoptera exigua* in oral toxicity bioassay at 95% confidence interval.

Insecticides	No.	Slope±SE	LC_50_(95% FL)	RT(95% CI)	χ^2^(*df*)	*P*
Chlor.	300	1.21±0.13	0.86(0.61–1.17)	1.00(0.64–1.57)	12.42(28)	0.99
Spinosad	350	1.63±0.15	4.48(3.56–5.62)	5.24(3.55–7.74)	9.34(33)	1.00
Pyr.	450	0.88±0.08	10.24(7.26–14.91)	11.98(7.44–19.29)	18.40(43)	0.99
Azad.+Pyr.	400	1.63±0.14	12.94(10.37–16.28)	15.13(10.25–22.34)	18.29(38)	0.99

FL, fiducial limit. *df*, degree of freedom. CI, confidence interval. No. = number of insects. LC_50_ = LC_50_ (95% FL) μg a.i./cup. Azadirachtin + pyrethrin = Azad. + Pyr., pyrethrin = Pyr. and chlorantraniliprole = Chlor. RT = >LC_50_ (least toxic)/< LC_50_ (most toxic).

The spinosad, in the oral toxicity bioassay, was the most toxic insecticide to *P*. *maculiventris*, followed by the pyrethrin, azadirachtin + pyrethrin and chlorantraniliprole, with relative toxicity of 1.00, 2.63, 2.87 and 10.89, respectively (Table 4).

**Table 4:** Relative toxicity (RT) of insecticides to the 3^rd^ instar spined soldier bug, *Podisus maculiventris* in oral toxicity bioassay at 95% confidence interval.

Insecticides	No.	Slope±SE	LC_50_(95% FL)	RT(95% CI)	χ^2^(*df*)	*P*
Spinosad	210	1.39±0.17	17.91(12.91–25.80)	1.00(0.62–1.61)	9.40(19)	0.97
Pyr.	210	1.33±0.17	47.07(33.27–66.37)	2.63(1.64–4.22)	3.17(19)	1.00
Azad.+Pyr.	210	0.95±0.15	51.42(32.47–81.98)	2.87(1.65–4.99)	5.03(19)	0.99
Chlor.	150	2.10±0.31	195.00(149.49–266.83)	10.89(7.05–16.82)	3.64(19)	0.99

FL, fiducial limit. *df*, degree of freedom. CI, confidence interval. No. = number of insects. LC_50_ = LC_50_ (95% FL) μg a.i./mL. Azadirachtin + pyrethrin = Azad. + Pyr., pyrethrin = Pyr. and chlorantraniliprole = Chlor. RT = >LC_50_ (least toxic)/< LC_50_ (most toxic).

### Behavioral response

**Fig 1:**
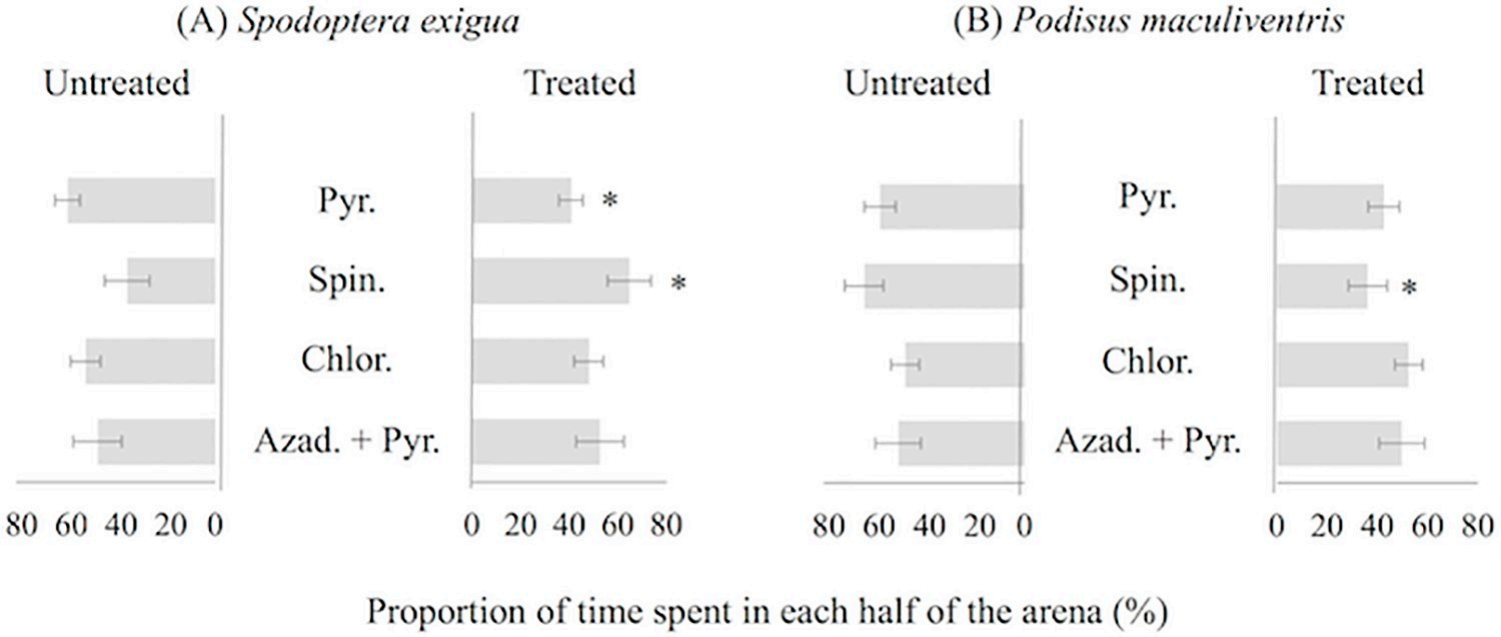
**Time proportion (mean ± SE) spent per 3**^**rd**^ **instar individual beet armyworm,** *Spodoptera exigua* **(A) and spined soldier bug,** *Podisus maculiventris* **(B) for 10-minute exposures to both the treated and untreated half of the Petri dishes (9 cm diameter) lined with filter paper treated with dried insecticide residue.** Asterisk in the bar indicates difference between Petri dishes treated or not with insecticide (paired Student’s *t* test at *P* < 0.05).

**Fig 2:**
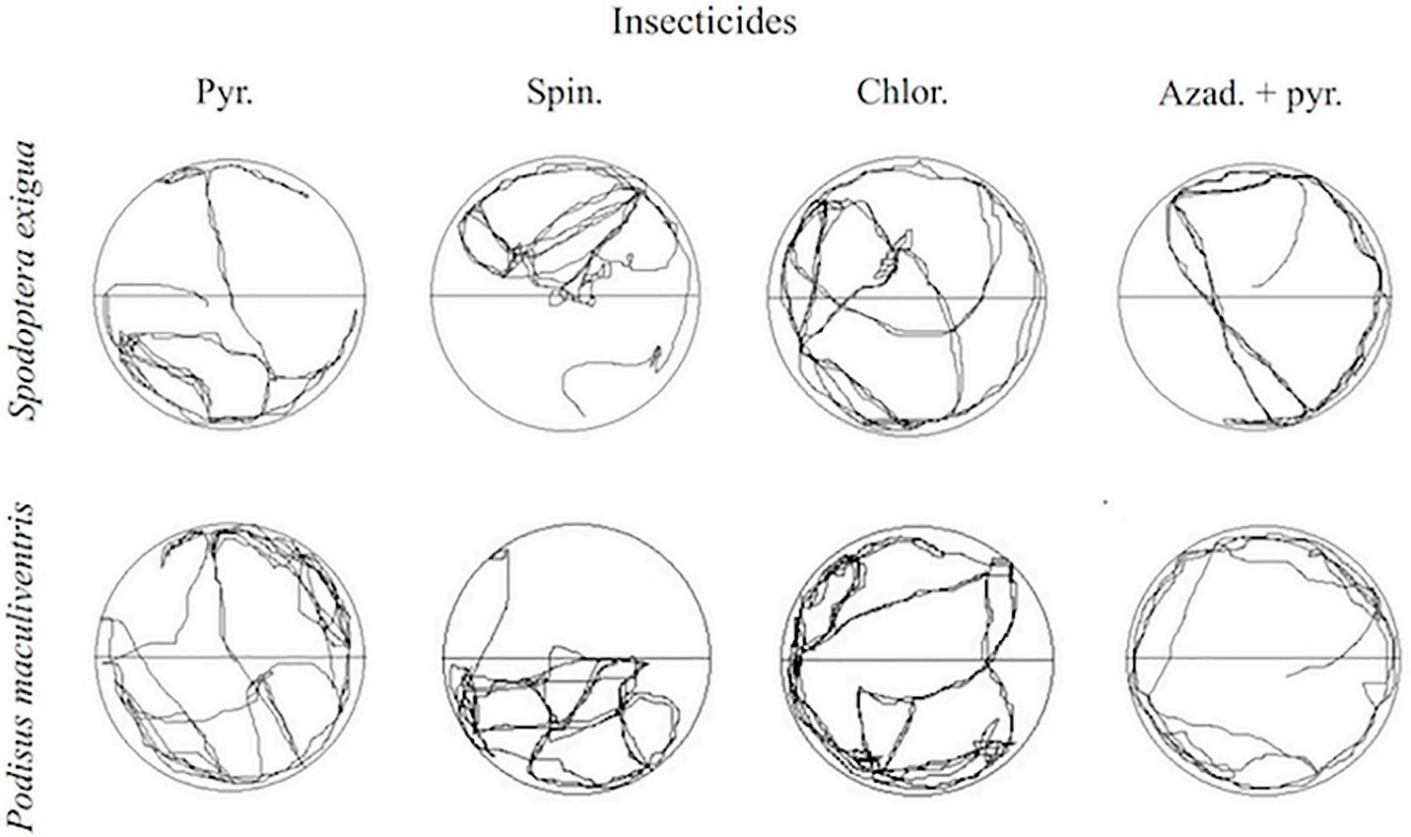
Tracks showing the movement of each 3^rd^ instar individual beet armyworm, *Spodoptera exigua* and spined soldier bug nymphs, *Podisus maculiventris*, for 10 minutes by Petri dishes lined with filter paper (9 cm diameter) with half impregnated with dry insecticide residues. Half lid of each Petri dish was treated.

The time proportion spent by *S*. *exigua* in the treated and untreated half of the dishes was similar for chlorantraniliprole and azadirachtin + pyrethrin; similar results were obtained for *P*. *maculiventris* with pyrethrin, chlorantraniliprole and azadirachtin + pyrethrin (*P* > 0.05). Nevertheless, behavior of avoidance to insecticides by *S*. *exigua* and *P*. *maculiventris* was detected. Behavioral avoidance to insecticide-treated surfaces was recognized through its two components–insecticide repellence (i.e., avoidance without contact) and insecticide irritability (i.e., avoidance after contact). The pyrethrin and spinosad reduced the time spent by *S*. *exigua* (t-test = 2.03; *df* = 34; *P* = 0.01) and by *P*. *maculiventris* (t-test = 2.05; *df* = 26; *P* = 0.02) in the treated half of the dish, respectively. The pest remained longer in the treated area with spinosad (t-test = 2.06; *df* = 26; *P* = 0.03) (Fig 1). Typical walking behavior of both species in areas partially treated with dry insecticide residues show that spinosad altered behavior causing irritability (avoidance after contact) for the predator and pyrethrin for the pest. The insecticide repellency (avoidance without contact) was not observed in the insect species tested. The walking speed and distance walked by *P*. *maculiventris* and *S*. *exigua* in treated or untreated parts of the dishes were similar (Fig 2).

## Discussion

Our study evaluated the effect of pesticides used in organic farm and a synthetic formulation on *S*. *litura* and its predator *P*. *maculiventris*. Three bioassays were carried out evaluating the direct contact to insect body or ingestion through artificial diet and drinking water of insecticides on the mortality and the behavior of the pest and its natural enemy. Spinosad and chlorantraniliprole were less toxic to *P*. *maculiventris* than to the pest, oppositely resulted for pyrethrin and azadirachtin + pyrethrin in contact toxicity bioassay. *Spodoptera exigua* was highly affected negatively by chlorantraniliprole in oral toxicity bioassay, followed by spinosad, pyrethrin and azadirachtin + pyrethrin. In oral toxicity bioassay, spinosad was the most toxic to *P*. *maculiventris*, followed by pyrethrin, azadirachtin + pyrethrin and chlorantraniliprole. The natural insecticides pyrethrin, azadirachtin + pyrethrin and spinosad were more toxic than the synthetic chlorantraniliprole to the predator.

The insecticides spinosad and chlorantraniliprole were more toxic to the pest than to the predator in this study, a condition essential for integrated pest management (IPM) programs [16,38]. Spinosad (Laser) caused 100% mortality under different temperature regimes to the generalist parasitoid *Bracon nigricans* (Szépligeti) (Hymenoptera: Braconidae) adults [39]. The chlorantraniliprole was slightly toxic to the predators *Podisus nigrispinus* (Dallas) and *Supputius cincticeps* (Stål) (Heteroptera: Pentatomidae), 72 h after to dry residues exposure [21]. This insecticide was also harmless according to the International Organization for Biological Control (IOBC) to the common eastern bumblebee, *Bombus impatiens* Cresson (Hymenoptera: Apidae) [40] and highly selective to the common green lacewing, *Chrysoperla carnea* (Stephens) (Neuroptera: Chrysopidae) [41]; the solitary aphid endoparasitoid, *Lysiphlebus testaceipes* (Cresson) (Hymenoptera: Braconidae) [42] and the Western predatory mite, *Galendromus occidentalis* (Nesbitt) (Acari: Phytoseiidae) [43]. The mortality of the mirid bug, *Macrolophus pygmaeus* (Rambur) (Hemiptera: Miridae) caused by chlorantraniliprole was lower than 25% and, therefore, classified as harmless [44]. This insecticide decreased the *M*. *pygmaeus* feeding on the plant, but did not affect the behavior of this insect. In addition, chlorantraniliprole did not affect the *M*. *pygmaeus* predation rate [44]. Chlorantraniliprole was more toxic to the pirate bugs, *Amphiareus constrictus* (Stål), *Blaptostethus pallescens* Poppius and to the minute pirate bug, *Orius tristicolor* (White) (Hemiptera: Anthocoridae) than to the tomato leafminer, *Tuta absoluta* (Meyrick) (Lepidoptera: Gelechiidae) and reduced the walking activity of the pest [45]. The high chlorantraniliprole toxicity is mainly due to the structure of its molecule with high affinity to lepidopteran ryanodine receptors [24,46].

The spinosad toxicity by contact or ingestion against *S*. *exigua* in this study agrees with reports for cruciferous lepidopteran-pests, including the diamondback moth, *Plutella xylostella* (L.) (Lepidoptera: Plutellidae), the small white, *Pieris rapae* (L.) (Lepidoptera: Pieridae) and the cabbage looper, *Trichoplusia ni* (Hübner) (Lepidoptera: Noctuidae) [47]. The spinosad has broad action spectrum, act on insects by a neural mechanism throught two effects (disruption of acetylcholine neurotransmission and as a γ-amino-butyric acid neurotransmitter agonist) and residual effects as a contact and stomach insecticide [48]. However, the spinosad selectivity for non-target species is debatable because in the present study it has been more toxic to *P*. *maculiventris* by ingestion in treated water than by contact with residues in the glass vial. A total of 71% of reports showed a lethal effect of spinosad for predatory insects in the laboratory and it is also toxic for some pollinators [49]. However, the irritability (i.e. avoidance after contact) showed by predatory stinkbugs to spinosad may increase these natural enemies survival [21].

The higher toxicity of insecticides used in organic farm (pyrethrin and azadiractin + pyrethrin) to *P*. *maculiventris* than to *S*. *exigua* in this study agrees with their knock-down effect on other insects [20]. The azadirachtin is the main insecticidal component of the neem plant, *Azadirachta indica* A. Juss. (Meliaceae) with widespread use against insect-pests [28]. However, the selectivity of azadiractin to predators is controversial and its safety for natural enemies has been questioned [12,50,51]. The *Chrysoperla externa* (Hagen) and *Ceraeochrysa cubana* (Hagen) (Neuroptera: Chrysopidae) mortality by azadirachtin was high (100%) [31] and this compound caused malformations to the predator *P*. *maculiventris* [12]. This compound reduced the survival of the harlequin ladybird, *Harmonia axyridis* (Pallas) (Coleoptera: Coccinellidae), from 3^rd^ instar to adulthood. The azadiractin also increased the larval stage of *H*. *axyridis* when applied on 1^st^ and 3^rd^ instar nymphs of this predator [52]. Compounds belonging to the tetranortriterpenoid group such as azadirachtin have exhibited a range of biological activities like insecticidal, insect antifeedant and growth regulating activity on insect pests [53].

The effect of the insecticides pyrethrin and spinosad on insect behavior in this study was expected because they are neurotoxic compounds whose sub-lethal impacts affect nerve interactions in these organisms [15,54,55]. The pyrethrin caused behavioral avoidance and reduced the time spent by *S*. *exigua* in the treated portion of the dish. A similar response, observed for *P*. *maculiventris* to spinosad, may be an adaptive behavior that reduces pest and predator exposure to toxic residues of this compound [56]. Pesticides, including herbicides and insecticides, can alter behavioral locomotion and reduce the efficiency of pest capture by predators and their mating [57–59]. The prolonged contact of *S*. *exigua* with the spinosad in the treated portion of the dish may indicate an arrestant effect of this compound, as reported for the European chafer, *Amphimallon majale* (Razoumowsky) (Coleoptera: Scarabaeidae) larvae with imidacloprid [60]. This neonicotinoid causes sublethal effects on natural enemies such as the predatory *P*. *nigrispinus* [61] and pollinators (e.g. bees) [62].

## Conclusion

The knowledge on risk associated with pesticides toward natural enemies is one of the key for IPM programs. The results obtained in the present study with the toxicity of the NOP compliant biopesticides Azera, Entrust and PyGanic Crop Protection EC 5.0 II, used in organic farm, and a synthetic formulation, Coragen, against *S*. *exigua* and *P*. *maculiventris* could improve IPM programs involving the use of *P*. *maculiventris* as natural enemy. The Entrust and especially the Coragen were more toxic to the pest than to the predator. However, Entrust, via ingestion of treated distilled water, was highly toxic to the predator, which could be negatively affected by this pesticide in the field by feeding on treated preys. The PyGanic Crop Protection EC 5.0 II and Azera were more toxic to this predator than to the pest. Coragen was less toxic than the botanicals PyGanic Crop Protection EC 5.0 II and Azera, and the microbial Entrust for the predator. Prior inclusion of biopesticides in IPM programs, their risk and the sub-lethal effects on non-target organisms should be assessed. The biopesticides Azera, Entrust and PyGanic Crop Protection EC 5.0 II are not recommended as insecticide options in areas with the predator *P*. *maculiventris*. Other natural enemy species which coexist with this predator in a same crop could be also affected negatively by these insecticides. The synthetic, Coragen showed potential selectivity for *P*. *maculiventris*. However, these results were obtained in laboratory conditions and the effect of this insecticide on pests and natural enemies is suggested to be tested in greenhouse and field. These tests are suggested to be conducted in a scenario involving multiple exposure routes (e.g. pesticide residue on plants and/or treated prey) and the combination of the pesticide fator with other stressors (e.g. temperature fluctuations).

## Supporting information

S1 File BAW- COR-TREATED DIET-10X5REP. (PDF)

S2 File BAW-AZE-TREATED DIET-10X5REP. (PDF)

S3 File BAW-AZE-VIALS-9X4REP. (PDF)

S4 File BAW-COR-VIALS-9X4REP. (PDF)

S5 File BAW-ENT-TREATED DIET-10X5REP. (PDF)

S6 File BAW-ENT-VIALS-9X4REP. (PDF)

S7 File BAW-PYG-TREATED DIET-10X5REP. (PDF)

S8 File BAW-PYG-VIALS-9X4REP. (PDF)

S9 File POD-AZE-ORALTOX-10X3REP. (PDF)

S10 File POD-AZE-VIALS-9X4REP. (PDF)

S11 File POD-COR-ORALTOX-10X3REP V2. (PDF)

S12 File POD-COR-ORALTOX-10X3REP. (PDF)

S13 File POD-ENT-ORALTOX-10X3REP. (PDF)

S14 File POD-ENT-VIALS-9X4REP. (PDF)

S15 File POD-PYG-ORALTOX-10X3REP. (PDF)

S16 File POD-PYG-VIALS-13X3REP. (PDF)

S17 File BAW POD INSECTICIDES SAS. (XLS)
