# Supplementary material for: Lethal and behavioral effects of synthetic and organic insecticides on Spodoptera exigua and its predator Podisus maculiventris
Source: PLoS One. 2018 Nov 8;13(11):e0206789. doi: 10.1371/journal.pone.0206789 (PMC6224277; doi:10.1371/journal.pone.0206789)
Supplement: S1 File — (PDF) [file pone.0206789.s001.pdf]

toxicidade de fenitrotion para populacao `SL

Obs	conc	total	mortos	mort	lconc
1	0.05	10	1	0.1	-1.30103
2	0.05	10	1	0.1	-1.30103
3	0.05	10	1	0.1	-1.30103
4	0.05	10	1	0.1	-1.30103
5	0.05	10	2	0.2	-1.30103
6	0.50	10	3	0.3	-0.30103
7	0.50	10	3	0.3	-0.30103
8	0.50	10	3	0.3	-0.30103
9	0.50	10	3	0.3	-0.30103
10	0.50	10	3	0.3	-0.30103
11	1.00	10	5	0.5	0.00000
12	1.00	10	5	0.5	0.00000
13	1.00	10	6	0.6	0.00000
14	1.00	10	5	0.5	0.00000
15	1.00	10	6	0.6	0.00000
16	2.50	10	6	0.6	0.39794
17	2.50	10	6	0.6	0.39794
18	2.50	10	7	0.7	0.39794
19	2.50	10	6	0.6	0.39794
20	2.50	10	7	0.7	0.39794
21	5.00	10	8	0.8	0.69897
22	5.00	10	8	0.8	0.69897
23	5.00	10	8	0.8	0.69897
24	5.00	10	8	0.8	0.69897
25	5.00	10	9	0.9	0.69897
26	10.00	10	10	1.0	1.00000
27	10.00	10	10	1.0	1.00000
28	10.00	10	9	0.9	1.00000
29	10.00	10	10	1.0	1.00000
30	10.00	10	10	1.0	1.00000

toxicidade de fenitrotion para populacao `SL

The Probit Procedure

Iteration History for Parameter Estimates				
**Iter**	**Ridge**	**Loglikelihood**	**Intercept**	**Log10(conc)**
**0**	0	-207.94415	0	0
**1**	0	-151.58751	0.0889754792	0.9473545799
**2**	0	-149.41429	0.0847791389	1.1894446873
**3**	0	-149.39926	0.0822811353	1.211979426
**4**	0	-149.39926	0.0822482254	1.2121738375
**5**	0	-149.39926	0.0822482254	1.2121738375

Model Information	
**Data Set**	WORK.UM
**Events Variable**	mortos
**Trials Variable**	total
**Number of Observations**	30
**Number of Events**	170
**Number of Trials**	300
**Name of Distribution**	Normal
**Log Likelihood**	-149.3992587

**Number of Observations Read**	30
**Number of Observations Used**	30
**Number of Events**	170
**Number of Trials**	300

Parameter Information	
**Parameter**	**Effect**
Intercept	Intercept
conc	conc

Last Evaluation of the Negative of the Gradient	
**Intercept**	**Log10(conc)**
2.3672604E-7	-8.275793E-7

Last Evaluation of the Negative of the Hessian		
	**Intercept**	**Log10(conc)**
**Intercept**	144.437967	13.708634043
**Log10(conc)**	13.708634043	59.135482556

Algorithm converged.

Goodness-of-Fit Tests				
**Statistic**	**Value**	**DF**	**Value/DF**	**Pr > ChiSq**
**Pearson Chi-Square**	12.4240	28	0.4437	0.9951
**L.R. Chi-Square**	15.3373	28	0.5478	0.9747

Note: Since the Pearson Chi-Square is small (p > 0.1000), fiducial limits will be calculated using a z value of 1.96

toxicidade de fenitrotion para populacao `SL

The Probit Procedure

Response-Covariate Profile	
Response Levels	2
Number of Covariate Values	30

Type III Analysis of Effects			
Effect	DF	Wald Chi-Square	Pr > ChiSq
Log10(conc)	1	84.9799	<.0001

Analysis of Maximum Likelihood Parameter Estimates							
Parameter	DF	Estimate	Standard Error	95% Confidence Limits		Chi-Square	Pr > ChiSq
Intercept	1	0.0822	0.0841	-0.0827	0.2472	0.96	0.3283
Log10(conc)	1	1.2122	0.1315	0.9544	1.4699	84.98	<.0001
_C_	0	0.0000	0.0000	0.0000	0.0000		

Estimated Covariance Matrix		
	Intercept	Log10(conc)
Intercept	0.007079	-0.001641
Log10(conc)	-0.001641	0.017291

Probit Model in Terms of Tolerance Distribution	
MU	SIGMA
-0.0678518	0.82496418

Estimated Covariance Matrix for Tolerance Parameters		
	MU	SIGMA
MU	0.005024	-0.001580
SIGMA	-0.001580	0.008009

toxicidade de fenitrotion para populacao `SL

The Probit Procedure

Probit Analysis on Log10(conc)			
Probability	Log10(conc)	95% Fiducial Limits	
0.01	-1.98701	-2.56919	-1.60196
0.02	-1.76212	-2.28588	-1.41420
0.03	-1.61944	-2.10642	-1.29478
0.04	-1.51211	-1.97161	-1.20477
0.05	-1.42480	-1.86209	-1.13140
0.06	-1.35048	-1.76899	-1.06884
0.07	-1.28533	-1.68745	-1.01389
0.08	-1.22699	-1.61454	-0.96460
0.09	-1.17393	-1.54830	-0.91969
0.10	-1.12509	-1.48741	-0.87828
0.15	-0.92287	-1.23624	-0.70587
0.20	-0.76216	-1.03807	-0.56740
0.25	-0.62428	-0.86953	-0.44713
0.30	-0.50046	-0.71979	-0.33751
0.35	-0.38573	-0.58287	-0.23409
0.40	-0.27685	-0.45511	-0.13380
0.45	-0.17152	-0.33406	-0.03421
0.50	-0.06785	-0.21793	0.06682
0.55	0.03581	-0.10530	0.17133
0.60	0.14115	0.00527	0.28141
0.65	0.25002	0.11542	0.39932
0.70	0.36476	0.22731	0.52776
0.75	0.48858	0.34399	0.67044
0.80	0.62646	0.47005	0.83319
0.85	0.78717	0.61326	1.02663
0.90	0.98938	0.78959	1.27387
0.91	1.03822	0.83172	1.33404
0.92	1.09128	0.87733	1.39958
0.93	1.14962	0.92731	1.47180
0.94	1.21478	0.98294	1.55266
0.95	1.28909	1.04618	1.64508
0.96	1.37640	1.12023	1.75391
0.97	1.48374	1.21096	1.88802
0.98	1.62642	1.33115	2.06671
0.99	1.85130	1.51982	2.34910

toxicidade de fenitrotion para populacao `SL

The Probit Procedure

Probit Analysis on conc			
Probability	conc	95% Fiducial Limits	
0.01	0.01030	0.00270	0.02501
0.02	0.01729	0.00518	0.03853
0.03	0.02402	0.00783	0.05072
0.04	0.03075	0.01068	0.06241
0.05	0.03760	0.01374	0.07389
0.06	0.04462	0.01702	0.08534
0.07	0.05184	0.02054	0.09685
0.08	0.05929	0.02429	0.10849
0.09	0.06700	0.02829	0.12031
0.10	0.07497	0.03255	0.13235
0.15	0.11943	0.05804	0.19685
0.20	0.17292	0.09161	0.27077
0.25	0.23753	0.13504	0.35716
0.30	0.31589	0.19064	0.45971
0.35	0.41141	0.26129	0.58332
0.40	0.52862	0.35066	0.73485
0.45	0.67372	0.46339	0.92425
0.50	0.85536	0.60543	1.16632
0.55	1.08596	0.78470	1.48364
0.60	1.38405	1.01221	1.91165
0.65	1.77838	1.30442	2.50795
0.70	2.31611	1.68775	3.37103
0.75	3.08019	2.20797	4.68208
0.80	4.23112	2.95157	6.81068
0.85	6.12588	4.10445	10.63240
0.90	9.75848	6.16019	18.78742
0.91	10.92001	6.78773	21.57956
0.92	12.33905	7.53935	25.09432
0.93	14.11312	8.45887	29.63489
0.94	16.39762	9.61487	35.69912
0.95	19.45779	11.12195	44.16538
0.96	23.79038	13.18961	56.74326
0.97	30.46039	16.25406	77.27087
0.98	42.30751	21.43612	116.60192
0.99	71.00711	33.09924	223.40693

NOTE: The above quantiles and fiducial limits refer to effects due to the independent variable and do not include any effect due to the natural threshold.

toxicidade de fenitrotion para populacao `SL

The REG Procedure

Model: MODEL1

Dependent Variable: mort

**Number of Observations Read**	30
**Number of Observations Used**	30

Analysis of Variance					
**Source**	**DF**	**Sum of Squares**	**Mean Square**	**F Value**	**Pr > F**
**Model**	1	2.42068	2.42068	364.44	<.0001
**Error**	28	0.18598	0.00664		
**Corrected Total**	29	2.60667			

**Root MSE**	0.08150	**R-Square**	0.9287
**Dependent Mean**	0.56667	**Adj R-Sq**	0.9261
**Coeff Var**	14.38233		

Parameter Estimates					
**Variable**	**DF**	**Parameter Estimate**	**Standard Error**	**t Value**	**Pr > |t|**
**Intercept**	1	0.53550	0.01497	35.77	<.0001
**lconc**	1	0.37794	0.01980	19.09	<.0001
