# Supplementary material for: Lethal and behavioral effects of synthetic and organic insecticides on Spodoptera exigua and its predator Podisus maculiventris
Source: PLoS One. 2018 Nov 8;13(11):e0206789. doi: 10.1371/journal.pone.0206789 (PMC6224277; doi:10.1371/journal.pone.0206789)
Supplement: S2 File — (PDF) [file pone.0206789.s002.pdf]

toxicidade de fenitrotion para populacao `SL

Obs	conc	total	mortos	mort	lconc
1	0.5	10	1	0.1	-0.30103
2	0.5	10	0	0.0	-0.30103
3	0.5	10	0	0.0	-0.30103
4	0.5	10	0	0.0	-0.30103
5	0.5	10	0	0.0	-0.30103
6	1.0	10	1	0.1	0.00000
7	1.0	10	0	0.0	0.00000
8	1.0	10	0	0.0	0.00000
9	1.0	10	1	0.1	0.00000
10	1.0	10	0	0.0	0.00000
11	2.5	10	1	0.1	0.39794
12	2.5	10	1	0.1	0.39794
13	2.5	10	1	0.1	0.39794
14	2.5	10	0	0.0	0.39794
15	2.5	10	1	0.1	0.39794
16	5.0	10	2	0.2	0.69897
17	5.0	10	2	0.2	0.69897
18	5.0	10	3	0.3	0.69897
19	5.0	10	3	0.3	0.69897
20	5.0	10	2	0.2	0.69897
21	10.0	10	5	0.5	1.00000
22	10.0	10	5	0.5	1.00000
23	10.0	10	5	0.5	1.00000
24	10.0	10	5	0.5	1.00000
25	10.0	10	5	0.5	1.00000
26	25.0	10	6	0.6	1.39794
27	25.0	10	6	0.6	1.39794
28	25.0	10	6	0.6	1.39794
29	25.0	10	7	0.7	1.39794
30	25.0	10	7	0.7	1.39794
31	50.0	10	8	0.8	1.69897
32	50.0	10	8	0.8	1.69897
33	50.0	10	8	0.8	1.69897
34	50.0	10	8	0.8	1.69897
35	50.0	10	9	0.9	1.69897
36	100.0	10	10	1.0	2.00000
37	100.0	10	9	0.9	2.00000
38	100.0	10	9	0.9	2.00000
39	100.0	10	9	0.9	2.00000
40	100.0	10	10	1.0	2.00000

toxicidade de fenitrotion para populacao `SL

The Probit Procedure

Iteration History for Parameter Estimates				
Iter	Ridge	Loglikelihood	Intercept	Log10(conc)
0	0	-277.25887	0	0
1	0	-168.19486	-1.173075997	1.0996759775
2	0	-158.87509	-1.674272216	1.5202196921
3	0	-158.50293	-1.803900109	1.6229699317
4	0	-158.50194	-1.811108448	1.6285722218
5	0	-158.50194	-1.811128925	1.6285880133
6	0	-158.50194	-1.811128925	1.6285880133

Model Information	
Data Set	WORK.UM
Events Variable	mortos
Trials Variable	total
Number of Observations	40
Number of Events	164
Number of Trials	400
Name of Distribution	Normal
Log Likelihood	-158.5019374

Number of Observations Read	40
Number of Observations Used	40
Number of Events	164
Number of Trials	400

Parameter Information	
Parameter	Effect
Intercept	Intercept
conc	conc

Last Evaluation of the Negative of the Gradient	
Intercept	Log10(conc)
4.8864257E-9	-1.712845E-9

Last Evaluation of the Negative of the Hessian		
	Intercept	Log10(conc)
Intercept	156.41436452	164.77154623
Log10(conc)	164.77154623	227.99451239

Algorithm converged.

Goodness-of-Fit Tests				
Statistic	Value	DF	Value/DF	Pr > ChiSq
Pearson Chi-Square	18.2877	38	0.4813	0.9971
L.R. Chi-Square	16.8647	38	0.4438	0.9988

Note: Since the Pearson Chi-Square is small (p > 0.1000), fiducial limits will be calculated using a z value of 1.96

toxicidade de fenitrotion para populacao `SL

The Probit Procedure

Response-Covariate Profile	
Response Levels	2
Number of Covariate Values	40

Type III Analysis of Effects			
Effect	DF	Wald Chi-Square	Pr > ChiSq
Log10(conc)	1	144.3362	<.0001

Analysis of Maximum Likelihood Parameter Estimates							
Parameter	DF	Estimate	Standard Error	95% Confidence Limits		Chi-Square	Pr > ChiSq
Intercept	1	-1.8111	0.1637	-2.1319	-1.4904	122.46	<.0001
Log10(conc)	1	1.6286	0.1356	1.3629	1.8943	144.34	<.0001
_C_	0	0.0000	0.0000	0.0000	0.0000		

Estimated Covariance Matrix		
	Intercept	Log10(conc)
Intercept	0.026785	-0.019358
Log10(conc)	-0.019358	0.018376

Probit Model in Terms of Tolerance Distribution	
MU	SIGMA
1.11208538	0.61402883

Estimated Covariance Matrix for Tolerance Parameters		
	MU	SIGMA
MU	0.002434	0.000250
SIGMA	0.000250	0.002612

toxicidade de fenitrotion para populacao `SL

The Probit Procedure

Probit Analysis on Log10(conc)			
Probability	Log10(conc)	95% Fiducial Limits	
0.01	-0.31636	-0.60325	-0.10438
0.02	-0.14898	-0.40574	0.04203
0.03	-0.04278	-0.28076	0.13526
0.04	0.03711	-0.18696	0.20561
0.05	0.10210	-0.11082	0.26300
0.06	0.15741	-0.04616	0.31198
0.07	0.20591	0.01043	0.35504
0.08	0.24933	0.06099	0.39370
0.09	0.28882	0.10687	0.42896
0.10	0.32518	0.14902	0.46150
0.15	0.47569	0.32245	0.59733
0.20	0.59531	0.45865	0.70691
0.25	0.69793	0.57392	0.80250
0.30	0.79009	0.67583	0.88994
0.35	0.87549	0.76863	0.97261
0.40	0.95652	0.85500	1.05275
0.45	1.03493	0.93684	1.13200
0.50	1.11209	1.01566	1.21172
0.55	1.18925	1.09280	1.29311
0.60	1.26765	1.16957	1.37744
0.65	1.34868	1.24739	1.46613
0.70	1.43408	1.32794	1.56104
0.75	1.52624	1.41350	1.66484
0.80	1.62887	1.50743	1.78176
0.85	1.74849	1.61556	1.91942
0.90	1.89899	1.75008	2.09415
0.91	1.93535	1.78237	2.13655
0.92	1.97484	1.81738	2.18268
0.93	2.01826	1.85580	2.23349
0.94	2.06676	1.89862	2.29032
0.95	2.12207	1.94735	2.35523
0.96	2.18706	2.00448	2.43162
0.97	2.26695	2.07456	2.52569
0.98	2.37315	2.16750	2.65097
0.99	2.54053	2.31355	2.84883

toxicidade de fenitrotion para populacao `SL

The Probit Procedure

Probit Analysis on conc			
Probability	conc	95% Fiducial Limits	
0.01	0.48266	0.24932	0.78637
0.02	0.70962	0.39288	1.10162
0.03	0.90620	0.52389	1.36540
0.04	1.08922	0.65019	1.60549
0.05	1.26502	0.77478	1.83230
0.06	1.43684	0.89917	2.05105
0.07	1.60660	1.02430	2.26486
0.08	1.77554	1.15076	2.47573
0.09	1.94457	1.27901	2.68510
0.10	2.11434	1.40937	2.89402
0.15	2.99010	2.10112	3.95663
0.20	3.93827	2.87507	5.09225
0.25	4.98803	3.74900	6.34603
0.30	6.16720	4.74059	7.76146
0.35	7.50736	5.86990	9.38888
0.40	9.04738	7.16137	11.29147
0.45	10.83741	8.64642	13.55199
0.50	12.94450	10.36719	16.28236
0.55	15.46127	12.38236	19.63874
0.60	18.52029	14.77654	23.84720
0.65	22.31944	17.67612	29.24997
0.70	27.16955	21.27865	36.39457
0.75	33.59244	25.91203	46.22078
0.80	42.54662	32.16873	60.50117
0.85	56.03835	41.26275	83.06565
0.90	79.24922	56.24422	124.20858
0.91	86.16832	60.58594	136.94647
0.92	94.37128	65.67241	152.29436
0.93	104.29503	71.74650	171.19389
0.94	116.61682	79.18066	195.12686
0.95	132.45640	88.58333	226.58563
0.96	153.83569	101.03792	270.16058
0.97	184.90425	118.73094	335.49864
0.98	236.12743	147.06032	447.67798
0.99	347.16030	205.84843	706.04806

NOTE: The above quantiles and fiducial limits refer to effects due to the independent variable and do not include any effect due to the natural threshold.

toxicidade de fenitrotion para populacao `SL

The REG Procedure

Model: MODEL1

Dependent Variable: mort

**Number of Observations Read**	40
**Number of Observations Used**	40

Analysis of Variance					
**Source**	**DF**	**Sum of Squares**	**Mean Square**	**F Value**	**Pr > F**
**Model**	1	4.46464	4.46464	582.28	<.0001
**Error**	38	0.29136	0.00767		
**Corrected Total**	39	4.75600			

**Root MSE**	0.08756	**R-Square**	0.9387
**Dependent Mean**	0.41000	**Adj R-Sq**	0.9371
**Coeff Var**	21.35707		

Parameter Estimates					
**Variable**	**DF**	**Parameter Estimate**	**Standard Error**	**t Value**	**Pr > |t|**
**Intercept**	1	0.03201	0.02091	1.53	0.1340
**lconc**	1	0.43871	0.01818	24.13	<.0001
