# Supplementary material for: Lethal and behavioral effects of synthetic and organic insecticides on Spodoptera exigua and its predator Podisus maculiventris
Source: PLoS One. 2018 Nov 8;13(11):e0206789. doi: 10.1371/journal.pone.0206789 (PMC6224277; doi:10.1371/journal.pone.0206789)
Supplement: S3 File — (PDF) [file pone.0206789.s003.pdf]

toxicidade de fenitrotion para populacao `SL

Obs	conc	total	mortos	mort	lconc
1	1.0	9	1	0.11111	0.00000
2	1.0	9	0	0.00000	0.00000
3	1.0	9	1	0.11111	0.00000
4	1.0	9	0	0.00000	0.00000
5	2.5	9	1	0.11111	0.39794
6	2.5	9	2	0.22222	0.39794
7	2.5	9	1	0.11111	0.39794
8	2.5	9	2	0.22222	0.39794
9	5.0	9	2	0.22222	0.69897
10	5.0	9	2	0.22222	0.69897
11	5.0	9	2	0.22222	0.69897
12	5.0	9	2	0.22222	0.69897
13	10.0	9	5	0.55556	1.00000
14	10.0	9	5	0.55556	1.00000
15	10.0	9	5	0.55556	1.00000
16	10.0	9	5	0.55556	1.00000
17	25.0	9	7	0.77778	1.39794
18	25.0	9	7	0.77778	1.39794
19	25.0	9	7	0.77778	1.39794
20	25.0	9	7	0.77778	1.39794
21	50.0	9	8	0.88889	1.69897
22	50.0	9	8	0.88889	1.69897
23	50.0	9	8	0.88889	1.69897
24	50.0	9	7	0.77778	1.69897
25	100.0	9	9	1.00000	2.00000
26	100.0	9	8	0.88889	2.00000
27	100.0	9	9	1.00000	2.00000
28	100.0	9	8	0.88889	2.00000

toxicidade de fenitrotion para populacao `SL

The Probit Procedure

Iteration History for Parameter Estimates				
Iter	Ridge	Loglikelihood	Intercept	Log10(conc)
0	0	-174.67309	0	0
1	0	-113.39832	-1.246946787	1.2423876554
2	0	-110.16299	-1.621256963	1.6159587392
3	0	-110.1228	-1.66902534	1.6638726061
4	0	-110.12279	-1.669764281	1.6646187878
5	0	-110.12279	-1.669764281	1.6646187878

Model Information	
Data Set	WORK.UM
Events Variable	mortos
Trials Variable	total
Number of Observations	28
Number of Events	129
Number of Trials	252
Name of Distribution	Normal
Log Likelihood	-110.1227864

Number of Observations Read	28
Number of Observations Used	28
Number of Events	129
Number of Trials	252

Parameter Information	
Parameter	Effect
Intercept	Intercept
conc	conc

Last Evaluation of the Negative of the Gradient	
Intercept	Log10(conc)
-6.632499E-7	-6.666328E-6

Last Evaluation of the Negative of the Hessian		
	Intercept	Log10(conc)
Intercept	108.38408378	110.35934895
Log10(conc)	110.35934895	145.92488535

Algorithm converged.

Goodness-of-Fit Tests				
Statistic	Value	DF	Value/DF	Pr > ChiSq
Pearson Chi-Square	8.5578	26	0.3291	0.9995
L.R. Chi-Square	9.3917	26	0.3612	0.9988

Note: Since the Pearson Chi-Square is small (p > 0.1000), fiducial limits will be calculated using a z value of 1.96

toxicidade de fenitrotion para populacao `SL

The Probit Procedure

Response-Covariate Profile	
Response Levels	2
Number of Covariate Values	28

Type III Analysis of Effects			
Effect	DF	Wald Chi-Square	Pr > ChiSq
Log10(conc)	1	92.9774	<.0001

Analysis of Maximum Likelihood Parameter Estimates							
Parameter	DF	Estimate	Standard Error	95% Confidence Limits		Chi-Square	Pr > ChiSq
Intercept	1	-1.6698	0.2003	-2.0624	-1.2772	69.49	<.0001
Log10(conc)	1	1.6646	0.1726	1.3263	2.0030	92.98	<.0001
_C_	0	0.0000	0.0000	0.0000	0.0000		

Estimated Covariance Matrix		
	Intercept	Log10(conc)
Intercept	0.040125	-0.030346
Log10(conc)	-0.030346	0.029802

Probit Model in Terms of Tolerance Distribution	
MU	SIGMA
1.00309109	0.60073814

Estimated Covariance Matrix for Tolerance Parameters		
	MU	SIGMA
MU	0.003332	-0.000098
SIGMA	-0.000098	0.003881

toxicidade de fenitrotion para populacao `SL

The Probit Procedure

Probit Analysis on Log10(conc)			
Probability	Log10(conc)	95% Fiducial Limits	
0.01	-0.39443	-0.77633	-0.13430
0.02	-0.23067	-0.57339	0.00440
0.03	-0.12677	-0.44496	0.09273
0.04	-0.04861	-0.34857	0.15939
0.05	0.01496	-0.27033	0.21378
0.06	0.06908	-0.20386	0.26021
0.07	0.11653	-0.14570	0.30103
0.08	0.15901	-0.09372	0.33769
0.09	0.19765	-0.04655	0.37112
0.10	0.23321	-0.00321	0.40198
0.15	0.38047	0.17514	0.53082
0.20	0.49750	0.31527	0.63484
0.25	0.59790	0.43389	0.72568
0.30	0.68806	0.53877	0.80890
0.35	0.77161	0.63419	0.88778
0.40	0.85090	0.72285	0.96452
0.45	0.92760	0.80660	1.04079
0.50	1.00309	0.88689	1.11799
0.55	1.07858	0.96497	1.19740
0.60	1.15529	1.04209	1.28030
0.65	1.23457	1.11961	1.36817
0.70	1.31812	1.19919	1.46290
0.75	1.40828	1.28303	1.56716
0.80	1.50869	1.37440	1.68524
0.85	1.62572	1.47889	1.82491
0.90	1.77297	1.60814	2.00285
0.91	1.80853	1.63907	2.04611
0.92	1.84717	1.67258	2.09321
0.93	1.88966	1.70931	2.14511
0.94	1.93710	1.75021	2.20320
0.95	1.99122	1.79672	2.26958
0.96	2.05479	1.85119	2.34775
0.97	2.13296	1.91793	2.44406
0.98	2.23686	2.00635	2.57240
0.99	2.40062	2.14515	2.77523

toxicidade de fenitrotion para populacao `SL

The Probit Procedure

Probit Analysis on conc			
**Probability**	**conc**	**95% Fiducial Limits**	
**0.01**	0.40324	0.16737	0.73401
**0.02**	0.58793	0.26706	1.01017
**0.03**	0.74684	0.35895	1.23802
**0.04**	0.89410	0.44816	1.44341
**0.05**	1.03506	0.53663	1.63599
**0.06**	1.17241	0.62537	1.82058
**0.07**	1.30776	0.71499	2.00001
**0.08**	1.44215	0.80589	2.17615
**0.09**	1.57633	0.89836	2.35027
**0.10**	1.71086	0.99263	2.52335
**0.15**	2.40141	1.49673	3.39484
**0.20**	3.14411	2.06666	4.31362
**0.25**	3.96186	2.71577	5.31712
**0.30**	4.87600	3.45757	6.44020
**0.35**	5.91037	4.30720	7.72285
**0.40**	7.09408	5.28264	9.21549
**0.45**	8.46450	6.40620	10.98478
**0.50**	10.07143	7.70701	13.12175
**0.55**	11.98342	9.22501	15.75421
**0.60**	14.29836	11.01756	19.06782
**0.65**	17.16200	13.17070	23.34394
**0.70**	20.80264	15.81941	29.03325
**0.75**	25.60253	19.18805	36.91095
**0.80**	32.26154	23.68103	48.44443
**0.85**	42.23925	30.12227	66.81997
**0.90**	59.28816	40.56378	100.65872
**0.91**	64.34781	43.55868	111.20188
**0.92**	70.33495	47.05232	123.93982
**0.93**	77.56308	51.20499	139.67198
**0.94**	86.51729	56.26157	159.65960
**0.95**	97.99804	62.62054	186.03019
**0.96**	113.44752	70.98805	222.71534
**0.97**	135.81744	82.78125	278.01063
**0.98**	172.52673	101.47360	373.59202
**0.99**	251.54576	139.68653	595.98094

NOTE: The above quantiles and fiducial limits refer to effects due to the independent variable and do not include any effect due to the natural threshold.

toxicidade de fenitrotion para populacao `SL

The REG Procedure

Model: MODEL1

Dependent Variable: mort

**Number of Observations Read**	28
**Number of Observations Used**	28

Analysis of Variance					
**Source**	**DF**	**Sum of Squares**	**Mean Square**	**F Value**	**Pr > F**
**Model**	1	3.04092	3.04092	445.85	<.0001
**Error**	26	0.17733	0.00682		
**Corrected Total**	27	3.21825			

**Root MSE**	0.08259	**R-Square**	0.9449
**Dependent Mean**	0.51190	**Adj R-Sq**	0.9428
**Coeff Var**	16.13316		

Parameter Estimates					
**Variable**	**DF**	**Parameter Estimate**	**Standard Error**	**t Value**	**Pr > |t|**
**Intercept**	1	0.00254	0.02873	0.09	0.9302
**lconc**	1	0.49564	0.02347	21.12	<.0001
