# Supplementary material for: Lethal and behavioral effects of synthetic and organic insecticides on Spodoptera exigua and its predator Podisus maculiventris
Source: PLoS One. 2018 Nov 8;13(11):e0206789. doi: 10.1371/journal.pone.0206789 (PMC6224277; doi:10.1371/journal.pone.0206789)
Supplement: S4 File — (PDF) [file pone.0206789.s004.pdf]

toxicidade de fenitrotion para populacao `SL

Obs	conc	total	mortos	mort	lconc
1	0.005	9	0	0.00000	-2.30103
2	0.005	9	1	0.11111	-2.30103
3	0.005	9	0	0.00000	-2.30103
4	0.005	9	0	0.00000	-2.30103
5	0.025	9	1	0.11111	-1.60206
6	0.025	9	1	0.11111	-1.60206
7	0.025	9	1	0.11111	-1.60206
8	0.025	9	1	0.11111	-1.60206
9	0.050	9	2	0.22222	-1.30103
10	0.050	9	3	0.33333	-1.30103
11	0.050	9	3	0.33333	-1.30103
12	0.050	9	2	0.22222	-1.30103
13	0.250	9	3	0.33333	-0.60206
14	0.250	9	3	0.33333	-0.60206
15	0.250	9	4	0.44444	-0.60206
16	0.250	9	4	0.44444	-0.60206
17	0.500	9	5	0.55556	-0.30103
18	0.500	9	5	0.55556	-0.30103
19	0.500	9	6	0.66667	-0.30103
20	0.500	9	5	0.55556	-0.30103
21	1.000	9	6	0.66667	0.00000
22	1.000	9	7	0.77778	0.00000
23	1.000	9	6	0.66667	0.00000
24	1.000	9	5	0.55556	0.00000
25	2.500	9	7	0.77778	0.39794
26	2.500	9	6	0.66667	0.39794
27	2.500	9	6	0.66667	0.39794
28	2.500	9	7	0.77778	0.39794
29	5.000	9	8	0.88889	0.69897
30	5.000	9	8	0.88889	0.69897
31	5.000	9	8	0.88889	0.69897
32	5.000	9	9	1.00000	0.69897

toxicidade de fenitrotion para populacao `SL

The Probit Procedure

Iteration History for Parameter Estimates				
Iter	Ridge	Loglikelihood	Intercept	Log10(conc)
0	0	-199.62639	0	0
1	0	-145.84372	0.369793329	0.7433209307
2	0	-143.31884	0.4273666657	0.9424580342
3	0	-143.29248	0.4312992898	0.9647368405
4	0	-143.29248	0.4313365556	0.9650183159
5	0	-143.29248	0.4313365556	0.9650183159

Model Information	
Data Set	WORK.UM
Events Variable	mortos
Trials Variable	total
Number of Observations	32
Number of Events	133
Number of Trials	288
Name of Distribution	Normal
Log Likelihood	-143.292477

Number of Observations Read	32
Number of Observations Used	32
Number of Events	133
Number of Trials	288

Parameter Information	
Parameter	Effect
Intercept	Intercept
conc	conc

Last Evaluation of the Negative of the Gradient	
Intercept	Log10(conc)
2.2362783E-6	-5.20456E-6

Last Evaluation of the Negative of the Hessian		
	Intercept	Log10(conc)
Intercept	138.30393306	-64.91961791
Log10(conc)	-64.91961791	121.2642327

Algorithm converged.

Goodness-of-Fit Tests				
Statistic	Value	DF	Value/DF	Pr > ChiSq
Pearson Chi-Square	10.0017	30	0.3334	0.9998
L.R. Chi-Square	11.3724	30	0.3791	0.9992

Note: Since the Pearson Chi-Square is small (p > 0.1000), fiducial limits will be calculated using a z value of 1.96

toxicidade de fenitrotion para populacao `SL

The Probit Procedure

Response-Covariate Profile	
Response Levels	2
Number of Covariate Values	32

Type III Analysis of Effects			
Effect	DF	Wald Chi-Square	Pr > ChiSq
Log10(conc)	1	84.5501	<.0001

Analysis of Maximum Likelihood Parameter Estimates							
Parameter	DF	Estimate	Standard Error	95% Confidence Limits		Chi-Square	Pr > ChiSq
Intercept	1	0.4313	0.0983	0.2387	0.6239	19.27	<.0001
Log10(conc)	1	0.9650	0.1049	0.7593	1.1707	84.55	<.0001
_C_	0	0.0000	0.0000	0.0000	0.0000		

Estimated Covariance Matrix		
	Intercept	Log10(conc)
Intercept	0.009657	0.005170
Log10(conc)	0.005170	0.011014

Probit Model in Terms of Tolerance Distribution	
MU	SIGMA
-0.4469724	1.03624976

Estimated Covariance Matrix for Tolerance Parameters		
	MU	SIGMA
MU	0.007770	0.000275
SIGMA	0.000275	0.012700

toxicidade de fenitrotion para populacao `SL

The Probit Procedure

Probit Analysis on Log10(conc)			
**Probability**	**Log10(conc)**	**95% Fiducial Limits**	
**0.01**	-2.8576	-3.5331	-2.4095
**0.02**	-2.5752	-3.1777	-2.1731
**0.03**	-2.3959	-2.9527	-2.0225
**0.04**	-2.2611	-2.7838	-1.9090
**0.05**	-2.1515	-2.6466	-1.8164
**0.06**	-2.0581	-2.5301	-1.7374
**0.07**	-1.9763	-2.4281	-1.6679
**0.08**	-1.9030	-2.3369	-1.6055
**0.09**	-1.8363	-2.2541	-1.5487
**0.10**	-1.7750	-2.1781	-1.4962
**0.15**	-1.5210	-1.8649	-1.2771
**0.20**	-1.3191	-1.6189	-1.1002
**0.25**	-1.1459	-1.4106	-0.9456
**0.30**	-0.9904	-1.2268	-0.8036
**0.35**	-0.8463	-1.0600	-0.6684
**0.40**	-0.7095	-0.9056	-0.5362
**0.45**	-0.5772	-0.7607	-0.4039
**0.50**	-0.4470	-0.6227	-0.2691
**0.55**	-0.3168	-0.4895	-0.1295
**0.60**	-0.1844	-0.3587	0.0170
**0.65**	-0.0477	-0.2279	0.1727
**0.70**	0.0964	-0.0939	0.3406
**0.75**	0.2520	0.0472	0.5255
**0.80**	0.4252	0.2010	0.7345
**0.85**	0.6270	0.3772	0.9812
**0.90**	0.8810	0.5958	1.2948
**0.91**	0.9424	0.6482	1.3710
**0.92**	1.0090	0.7049	1.4539
**0.93**	1.0823	0.7672	1.5452
**0.94**	1.1642	0.8366	1.6473
**0.95**	1.2575	0.9155	1.7639
**0.96**	1.3672	1.0080	1.9012
**0.97**	1.5020	1.1215	2.0702
**0.98**	1.6812	1.2719	2.2953
**0.99**	1.9637	1.5082	2.6509

toxicidade de fenitrotion para populacao `SL

The Probit Procedure

Probit Analysis on conc			
**Probability**	**conc**	**95% Fiducial Limits**	
**0.01**	0.00139	0.0002930	0.00389
**0.02**	0.00266	0.0006641	0.00671
**0.03**	0.00402	0.00111	0.00949
**0.04**	0.00548	0.00165	0.01233
**0.05**	0.00706	0.00226	0.01526
**0.06**	0.00875	0.00295	0.01831
**0.07**	0.01056	0.00373	0.02148
**0.08**	0.01250	0.00460	0.02480
**0.09**	0.01458	0.00557	0.02827
**0.10**	0.01679	0.00664	0.03190
**0.15**	0.03013	0.01365	0.05283
**0.20**	0.04796	0.02405	0.07939
**0.25**	0.07146	0.03885	0.11335
**0.30**	0.10224	0.05932	0.15719
**0.35**	0.14248	0.08710	0.21457
**0.40**	0.19521	0.12427	0.29093
**0.45**	0.26473	0.17349	0.39453
**0.50**	0.35730	0.23837	0.53818
**0.55**	0.48222	0.32396	0.74221
**0.60**	0.65397	0.43778	1.03989
**0.65**	0.89602	0.59171	1.48823
**0.70**	1.24864	0.80555	2.19102
**0.75**	1.78635	1.11469	3.35313
**0.80**	2.66169	1.58856	5.42585
**0.85**	4.23674	2.38367	9.57613
**0.90**	7.60388	3.94251	19.71740
**0.91**	8.75759	4.44792	23.49655
**0.92**	10.21016	5.06904	28.43650
**0.93**	12.08692	5.85051	35.08731
**0.94**	14.59356	6.86389	44.38705
**0.95**	18.09284	8.23197	58.06301
**0.96**	23.29033	10.18625	79.64643
**0.97**	31.76871	13.22691	117.54501
**0.98**	47.99814	18.70072	197.38552
**0.99**	91.98246	32.22356	447.56012

NOTE: The above quantiles and fiducial limits refer to effects due to the independent variable and do not include any effect due to the natural threshold.

toxicidade de fenitrotion para populacao `SL

The REG Procedure

Model: MODEL1

Dependent Variable: mort

**Number of Observations Read**	32
**Number of Observations Used**	32

Analysis of Variance					
**Source**	**DF**	**Sum of Squares**	**Mean Square**	**F Value**	**Pr > F**
**Model**	1	2.64379	2.64379	456.56	<.0001
**Error**	30	0.17372	0.00579		
**Corrected Total**	31	2.81752			

**Root MSE**	0.07610	**R-Square**	0.9383
**Dependent Mean**	0.46181	**Adj R-Sq**	0.9363
**Coeff Var**	16.47812		

Parameter Estimates					
**Variable**	**DF**	**Parameter Estimate**	**Standard Error**	**t Value**	**Pr > |t|**
**Intercept**	1	0.64753	0.01602	40.43	<.0001
**lconc**	1	0.29654	0.01388	21.37	<.0001
