# Supplementary material for: Lethal and behavioral effects of synthetic and organic insecticides on Spodoptera exigua and its predator Podisus maculiventris
Source: PLoS One. 2018 Nov 8;13(11):e0206789. doi: 10.1371/journal.pone.0206789 (PMC6224277; doi:10.1371/journal.pone.0206789)
Supplement: S5 File — (PDF) [file pone.0206789.s005.pdf]

toxicidade de fenitrotion para populacao `SL

Obs	conc	total	mortos	mort	lconc
1	0.5	10	0	0.0	-0.30103
2	0.5	10	0	0.0	-0.30103
3	0.5	10	0	0.0	-0.30103
4	0.5	10	1	0.1	-0.30103
5	0.5	10	1	0.1	-0.30103
6	1.0	10	2	0.2	0.00000
7	1.0	10	2	0.2	0.00000
8	1.0	10	2	0.2	0.00000
9	1.0	10	1	0.1	0.00000
10	1.0	10	1	0.1	0.00000
11	2.5	10	3	0.3	0.39794
12	2.5	10	3	0.3	0.39794
13	2.5	10	3	0.3	0.39794
14	2.5	10	4	0.4	0.39794
15	2.5	10	4	0.4	0.39794
16	5.0	10	6	0.6	0.69897
17	5.0	10	6	0.6	0.69897
18	5.0	10	6	0.6	0.69897
19	5.0	10	6	0.6	0.69897
20	5.0	10	5	0.5	0.69897
21	10.0	10	7	0.7	1.00000
22	10.0	10	7	0.7	1.00000
23	10.0	10	7	0.7	1.00000
24	10.0	10	7	0.7	1.00000
25	10.0	10	6	0.6	1.00000
26	25.0	10	9	0.9	1.39794
27	25.0	10	8	0.8	1.39794
28	25.0	10	9	0.9	1.39794
29	25.0	10	9	0.9	1.39794
30	25.0	10	9	0.9	1.39794
31	50.0	10	10	1.0	1.69897
32	50.0	10	10	1.0	1.69897
33	50.0	10	9	0.9	1.69897
34	50.0	10	9	0.9	1.69897
35	50.0	10	10	1.0	1.69897

toxicidade de fenitrotion para populacao `SL

The Probit Procedure

Iteration History for Parameter Estimates				
Iter	Ridge	Loglikelihood	Intercept	Log10(conc)
0	0	-242.60151	0	0
1	0	-159.78896	-0.79617941	1.2107987046
2	0	-155.26629	-1.031878542	1.5813522281
3	0	-155.20408	-1.063031118	1.6316050328
4	0	-155.20406	-1.063571903	1.6324900908
5	0	-155.20406	-1.063571903	1.6324900908

Model Information	
Data Set	WORK.UM
Events Variable	mortos
Trials Variable	total
Number of Observations	35
Number of Events	182
Number of Trials	350
Name of Distribution	Normal
Log Likelihood	-155.2040617

Number of Observations Read	35
Number of Observations Used	35
Number of Events	182
Number of Trials	350

Parameter Information	
Parameter	Effect
Intercept	Intercept
conc	conc

Last Evaluation of the Negative of the Gradient	
Intercept	Log10(conc)
-2.770881E-6	-0.000014503

Last Evaluation of the Negative of the Hessian		
	Intercept	Log10(conc)
Intercept	151.99066036	102.09168832
Log10(conc)	102.09168832	115.19516224

Algorithm converged.

Goodness-of-Fit Tests				
Statistic	Value	DF	Value/DF	Pr > ChiSq
Pearson Chi-Square	9.3391	33	0.2830	1.0000
L.R. Chi-Square	11.7538	33	0.3562	0.9998

Note: Since the Pearson Chi-Square is small (p > 0.1000), fiducial limits will be calculated using a z value of 1.96

toxicidade de fenitrotion para populacao `SL

The Probit Procedure

Response-Covariate Profile	
Response Levels	2
Number of Covariate Values	35

Type III Analysis of Effects			
Effect	DF	Wald Chi-Square	Pr > ChiSq
Log10(conc)	1	124.2447	<.0001

Analysis of Maximum Likelihood Parameter Estimates							
Parameter	DF	Estimate	Standard Error	95% Confidence Limits		Chi-Square	Pr > ChiSq
Intercept	1	-1.0636	0.1275	-1.3135	-0.8137	69.58	<.0001
Log10(conc)	1	1.6325	0.1465	1.3454	1.9195	124.24	<.0001
_C_	0	0.0000	0.0000	0.0000	0.0000		

Estimated Covariance Matrix		
	Intercept	Log10(conc)
Intercept	0.016257	-0.014408
Log10(conc)	-0.014408	0.021450

Probit Model in Terms of Tolerance Distribution	
MU	SIGMA
0.65150282	0.61256115

Estimated Covariance Matrix for Tolerance Parameters		
	MU	SIGMA
MU	0.002472	-0.000100
SIGMA	-0.000100	0.003020

toxicidade de fenitrotion para populacao `SL

The Probit Procedure

Probit Analysis on Log10(conc)			
Probability	Log10(conc)	95% Fiducial Limits	
**0.01**	-0.77353	-1.09991	-0.53937
**0.02**	-0.60654	-0.89947	-0.39518
**0.03**	-0.50060	-0.77259	-0.30341
**0.04**	-0.42090	-0.67732	-0.23420
**0.05**	-0.35607	-0.59996	-0.17776
**0.06**	-0.30089	-0.53423	-0.12961
**0.07**	-0.25251	-0.47669	-0.08730
**0.08**	-0.20919	-0.42527	-0.04932
**0.09**	-0.16979	-0.37857	-0.01471
**0.10**	-0.13353	-0.33566	0.01723
**0.15**	0.01662	-0.15892	0.15037
**0.20**	0.13596	-0.01983	0.25756
**0.25**	0.23834	0.09814	0.35088
**0.30**	0.33028	0.20266	0.43610
**0.35**	0.41547	0.29799	0.51660
**0.40**	0.49631	0.38680	0.59463
**0.45**	0.57453	0.47094	0.67191
**0.50**	0.65150	0.55187	0.74985
**0.55**	0.72848	0.63083	0.82975
**0.60**	0.80669	0.70909	0.91291
**0.65**	0.88754	0.78803	1.00082
**0.70**	0.97273	0.86933	1.09534
**0.75**	1.06467	0.95526	1.19915
**0.80**	1.16705	1.04919	1.31651
**0.85**	1.28638	1.15691	1.45508
**0.90**	1.43653	1.29051	1.63136
**0.91**	1.47280	1.32253	1.67418
**0.92**	1.51220	1.35723	1.72079
**0.93**	1.55552	1.39529	1.77214
**0.94**	1.60390	1.43769	1.82959
**0.95**	1.65908	1.48592	1.89523
**0.96**	1.72391	1.54245	1.97250
**0.97**	1.80360	1.61175	2.06768
**0.98**	1.90955	1.70362	2.19447
**0.99**	2.07653	1.84793	2.39478

toxicidade de fenitrotion para populacao `SL

The Probit Procedure

Probit Analysis on conc			
**Probability**	**conc**	**95% Fiducial Limits**	
**0.01**	0.16845	0.07945	0.28882
**0.02**	0.24743	0.12604	0.40255
**0.03**	0.31579	0.16882	0.49726
**0.04**	0.37940	0.21022	0.58318
**0.05**	0.44048	0.25121	0.66411
**0.06**	0.50016	0.29226	0.74197
**0.07**	0.55910	0.33366	0.81790
**0.08**	0.61775	0.37561	0.89264
**0.09**	0.67641	0.41824	0.96670
**0.10**	0.73532	0.46168	1.04047
**0.15**	1.03902	0.69356	1.41373
**0.20**	1.36760	0.95537	1.80950
**0.25**	1.73116	1.25355	2.24326
**0.30**	2.13932	1.59463	2.72963
**0.35**	2.60298	1.98605	3.28550
**0.40**	3.13554	2.43668	3.93219
**0.45**	3.75429	2.95762	4.69799
**0.50**	4.48232	3.56340	5.62150
**0.55**	5.35153	4.27395	6.75694
**0.60**	6.40757	5.11785	8.18302
**0.65**	7.71854	6.13799	10.01883
**0.70**	9.39140	7.40169	12.45484
**0.75**	11.60564	9.02116	15.81799
**0.80**	14.69086	11.19935	20.72574
**0.85**	19.33667	14.35192	28.51519
**0.90**	27.32320	19.52122	42.79176
**0.91**	29.70279	21.01503	47.22617
**0.92**	32.52333	22.76305	52.57652
**0.93**	35.93479	24.84789	59.17481
**0.94**	40.16953	27.39613	67.54416
**0.95**	45.61170	30.61429	78.56567
**0.96**	52.95477	34.86975	93.86478
**0.97**	63.62150	40.90284	116.86411
**0.98**	81.19880	50.53805	156.48330
**0.99**	119.27053	70.45771	248.18769

NOTE: The above quantiles and fiducial limits refer to effects due to the independent variable and do not include any effect due to the natural threshold.

toxicidade de fenitrotion para populacao `SL

The REG Procedure

Model: MODEL1

Dependent Variable: mort

**Number of Observations Read**	35
**Number of Observations Used**	35

Analysis of Variance					
Source	DF	Sum of Squares	Mean Square	F Value	Pr > F
Model	1	3.68464	3.68464	1091.90	<.0001
Error	33	0.11136	0.00337		
Corrected Total	34	3.79600			

**Root MSE**	0.05809	**R-Square**	0.9707
**Dependent Mean**	0.52000	**Adj R-Sq**	0.9698
**Coeff Var**	11.17128		

Parameter Estimates					
Variable	DF	Parameter Estimate	Standard Error	t Value	Pr > |t|
Intercept	1	0.18237	0.01417	12.87	<.0001
lconc	1	0.48304	0.01462	33.04	<.0001
