# Supplementary material for: Lethal and behavioral effects of synthetic and organic insecticides on Spodoptera exigua and its predator Podisus maculiventris
Source: PLoS One. 2018 Nov 8;13(11):e0206789. doi: 10.1371/journal.pone.0206789 (PMC6224277; doi:10.1371/journal.pone.0206789)
Supplement: S6 File — (PDF) [file pone.0206789.s006.pdf]

toxicidade de fenitrotion para populacao `SL

Obs	conc	total	mortos	mort	lconc
1	0.5	9	1	0.11111	-0.30103
2	0.5	9	0	0.00000	-0.30103
3	0.5	9	1	0.11111	-0.30103
4	0.5	9	0	0.00000	-0.30103
5	1.0	9	2	0.22222	0.00000
6	1.0	9	1	0.11111	0.00000
7	1.0	9	2	0.22222	0.00000
8	1.0	9	1	0.11111	0.00000
9	2.5	9	4	0.44444	0.39794
10	2.5	9	4	0.44444	0.39794
11	2.5	9	3	0.33333	0.39794
12	2.5	9	3	0.33333	0.39794
13	5.0	9	5	0.55556	0.69897
14	5.0	9	5	0.55556	0.69897
15	5.0	9	5	0.55556	0.69897
16	5.0	9	6	0.66667	0.69897
17	10.0	9	7	0.77778	1.00000
18	10.0	9	7	0.77778	1.00000
19	10.0	9	7	0.77778	1.00000
20	10.0	9	7	0.77778	1.00000
21	25.0	9	8	0.88889	1.39794
22	25.0	9	8	0.88889	1.39794
23	25.0	9	8	0.88889	1.39794
24	25.0	9	7	0.77778	1.39794
25	50.0	9	9	1.00000	1.69897
26	50.0	9	9	1.00000	1.69897
27	50.0	9	9	1.00000	1.69897
28	50.0	9	8	0.88889	1.69897

toxicidade de fenitrotion para populacao `SL

The Probit Procedure

Iteration History for Parameter Estimates				
**Iter**	**Ridge**	**Loglikelihood**	**Intercept**	**Log10(conc)**
**0**	0	-174.67309	0	0
**1**	0	-114.6396	-0.733301587	1.2056567454
**2**	0	-111.25481	-0.941448946	1.5782609686
**3**	0	-111.20451	-0.968817264	1.6305930829
**4**	0	-111.20449	-0.969316925	1.6315839863
**5**	0	-111.20449	-0.969316925	1.6315839863

Model Information	
**Data Set**	WORK.UM
**Events Variable**	mortos
**Trials Variable**	total
**Number of Observations**	28
**Number of Events**	137
**Number of Trials**	252
**Name of Distribution**	Normal
**Log Likelihood**	-111.2044918

**Number of Observations Read**	28
**Number of Observations Used**	28
**Number of Events**	137
**Number of Trials**	252

Parameter Information	
**Parameter**	**Effect**
Intercept	Intercept
conc	conc

Last Evaluation of the Negative of the Gradient	
**Intercept**	**Log10(conc)**
-5.57714E-6	-0.000015374

Last Evaluation of the Negative of the Hessian		
	**Intercept**	**Log10(conc)**
**Intercept**	108.99198086	69.414370219
**Log10(conc)**	69.414370219	77.7325971

Algorithm converged.

Goodness-of-Fit Tests				
**Statistic**	**Value**	**DF**	**Value/DF**	**Pr > ChiSq**
**Pearson Chi-Square**	7.9515	26	0.3058	0.9997
**L.R. Chi-Square**	9.2359	26	0.3552	0.9990

Note: Since the Pearson Chi-Square is small (p > 0.1000), fiducial limits will be calculated using a z value of 1.96

toxicidade de fenitrotion para populacao `SL

The Probit Procedure

Response-Covariate Profile	
Response Levels	2
Number of Covariate Values	28

Type III Analysis of Effects			
Effect	DF	Wald Chi-Square	Pr > ChiSq
Log10(conc)	1	89.2438	<.0001

Analysis of Maximum Likelihood Parameter Estimates							
Parameter	DF	Estimate	Standard Error	95% Confidence Limits		Chi-Square	Pr > ChiSq
Intercept	1	-0.9693	0.1459	-1.2552	-0.6834	44.17	<.0001
Log10(conc)	1	1.6316	0.1727	1.2931	1.9701	89.24	<.0001
_C_	0	0.0000	0.0000	0.0000	0.0000		

Estimated Covariance Matrix		
	Intercept	Log10(conc)
Intercept	0.021274	-0.018997
Log10(conc)	-0.018997	0.029829

Probit Model in Terms of Tolerance Distribution	
MU	SIGMA
0.59409564	0.61290133

Estimated Covariance Matrix for Tolerance Parameters		
	MU	SIGMA
MU	0.003467	-0.000294
SIGMA	-0.000294	0.004209

toxicidade de fenitrotion para populacao `SL

The Probit Procedure

Probit Analysis on Log10(conc)			
Probability	Log10(conc)	95% Fiducial Limits	
0.01	-0.83173	-1.23722	-0.55835
0.02	-0.66465	-1.02890	-0.41748
0.03	-0.55865	-0.89706	-0.32779
0.04	-0.47890	-0.79808	-0.26010
0.05	-0.41404	-0.71772	-0.20490
0.06	-0.35883	-0.64945	-0.15778
0.07	-0.31042	-0.58970	-0.11635
0.08	-0.26707	-0.53630	-0.07917
0.09	-0.22765	-0.48783	-0.04526
0.10	-0.19137	-0.44329	-0.01396
0.15	-0.04114	-0.25990	0.11663
0.20	0.07826	-0.11569	0.22197
0.25	0.18070	0.00650	0.31386
0.30	0.27269	0.11464	0.39798
0.35	0.35793	0.21312	0.47765
0.40	0.43882	0.30469	0.55513
0.45	0.51708	0.39123	0.63215
0.50	0.59410	0.47418	0.71016
0.55	0.67111	0.55480	0.79051
0.60	0.74937	0.63431	0.87456
0.65	0.83026	0.71408	0.96383
0.70	0.91550	0.79581	1.06026
0.75	1.00749	0.88171	1.16661
0.80	1.10993	0.97515	1.28726
0.85	1.22933	1.08181	1.43014
0.90	1.37956	1.21356	1.61237
0.91	1.41585	1.24508	1.65669
0.92	1.45527	1.27920	1.70496
0.93	1.49861	1.31660	1.75814
0.94	1.54702	1.35824	1.81768
0.95	1.60223	1.40557	1.88573
0.96	1.66709	1.46100	1.96587
0.97	1.74684	1.52891	2.06462
0.98	1.85284	1.61886	2.19621
0.99	2.01992	1.76003	2.40422

toxicidade de fenitrotion para populacao `SL

The Probit Procedure

Probit Analysis on conc			
Probability	conc	95% Fiducial Limits	
0.01	0.14732	0.05791	0.27647
0.02	0.21645	0.09356	0.38240
0.03	0.27628	0.12675	0.47013
0.04	0.33197	0.15919	0.54941
0.05	0.38545	0.19155	0.62388
0.06	0.43770	0.22415	0.69538
0.07	0.48931	0.25721	0.76497
0.08	0.54066	0.29087	0.83336
0.09	0.59203	0.32522	0.90104
0.10	0.64362	0.36034	0.96836
0.15	0.90963	0.54967	1.30807
0.20	1.19747	0.76614	1.66712
0.25	1.51600	1.01509	2.05996
0.30	1.87366	1.30209	2.50021
0.35	2.27999	1.63352	3.00362
0.40	2.74675	2.01694	3.59027
0.45	3.28910	2.46168	4.28693
0.50	3.92731	2.97977	5.13051
0.55	4.68936	3.58753	6.17322
0.60	5.61529	4.30831	7.49132
0.65	6.76486	5.17708	9.20090
0.70	8.23193	6.24893	11.48849
0.75	10.17399	7.61576	14.67610
0.80	12.88031	9.44382	19.37588
0.85	16.95614	12.07286	26.92413
0.90	23.96405	16.35171	40.96100
0.91	26.05231	17.58233	45.36212
0.92	28.52764	19.01954	50.69385
0.93	31.52172	20.73005	57.29839
0.94	35.23858	22.81585	65.71704
0.95	40.01553	25.44312	76.86550
0.96	46.46153	28.90675	92.44161
0.97	55.82600	33.79956	116.04214
0.98	71.25922	41.57761	157.11317
0.99	104.69293	57.54839	253.64244

NOTE: The above quantiles and fiducial limits refer to effects due to the independent variable and do not include any effect due to the natural threshold.

toxicidade de fenitrotion para populacao `SL

The REG Procedure

Model: MODEL1

Dependent Variable: mort

**Number of Observations Read**	28
**Number of Observations Used**	28

Analysis of Variance					
**Source**	**DF**	**Sum of Squares**	**Mean Square**	**F Value**	**Pr > F**
**Model**	1	2.92273	2.92273	619.40	<.0001
**Error**	26	0.12269	0.00472		
**Corrected Total**	27	3.04541			

**Root MSE**	0.06869	**R-Square**	0.9597
**Dependent Mean**	0.54365	**Adj R-Sq**	0.9582
**Coeff Var**	12.63541		

Parameter Estimates					
**Variable**	**DF**	**Parameter Estimate**	**Standard Error**	**t Value**	**Pr > |t|**
**Intercept**	1	0.20745	0.01874	11.07	<.0001
**lconc**	1	0.48099	0.01933	24.89	<.0001
