# Supplementary material for: Lethal and behavioral effects of synthetic and organic insecticides on Spodoptera exigua and its predator Podisus maculiventris
Source: PLoS One. 2018 Nov 8;13(11):e0206789. doi: 10.1371/journal.pone.0206789 (PMC6224277; doi:10.1371/journal.pone.0206789)
Supplement: S7 File — (PDF) [file pone.0206789.s007.pdf]

toxicidade de fenitrotion para populacao `SL

Obs	conc	total	mortos	mort	lconc
1	0.05	10	1	0.1	-1.30103
2	0.05	10	0	0.0	-1.30103
3	0.05	10	0	0.0	-1.30103
4	0.05	10	1	0.1	-1.30103
5	0.05	10	0	0.0	-1.30103
6	0.50	10	1	0.1	-0.30103
7	0.50	10	1	0.1	-0.30103
8	0.50	10	2	0.2	-0.30103
9	0.50	10	2	0.2	-0.30103
10	0.50	10	1	0.1	-0.30103
11	1.00	10	2	0.2	0.00000
12	1.00	10	2	0.2	0.00000
13	1.00	10	2	0.2	0.00000
14	1.00	10	2	0.2	0.00000
15	1.00	10	3	0.3	0.00000
16	2.50	10	3	0.3	0.39794
17	2.50	10	3	0.3	0.39794
18	2.50	10	3	0.3	0.39794
19	2.50	10	2	0.2	0.39794
20	2.50	10	2	0.2	0.39794
21	5.00	10	4	0.4	0.69897
22	5.00	10	4	0.4	0.69897
23	5.00	10	3	0.3	0.69897
24	5.00	10	3	0.3	0.69897
25	5.00	10	4	0.4	0.69897
26	10.00	10	4	0.4	1.00000
27	10.00	10	5	0.5	1.00000
28	10.00	10	4	0.4	1.00000
29	10.00	10	4	0.4	1.00000
30	10.00	10	5	0.5	1.00000
31	25.00	10	5	0.5	1.39794
32	25.00	10	5	0.5	1.39794
33	25.00	10	5	0.5	1.39794
34	25.00	10	5	0.5	1.39794
35	25.00	10	6	0.6	1.39794
36	50.00	10	8	0.8	1.69897
37	50.00	10	8	0.8	1.69897
38	50.00	10	8	0.8	1.69897
39	50.00	10	8	0.8	1.69897
40	50.00	10	7	0.7	1.69897
41	100.00	10	9	0.9	2.00000
42	100.00	10	9	0.9	2.00000
43	100.00	10	9	0.9	2.00000
44	100.00	10	9	0.9	2.00000
45	100.00	10	9	0.9	2.00000

toxicidade de fenitrotion para populacao `SL

The Probit Procedure

Iteration History for Parameter Estimates				
**Iter**	**Ridge**	**Loglikelihood**	**Intercept**	**Log10(conc)**
**0**	0	-311.91623	0	0
**1**	0	-236.78353	-0.637437289	0.6494141075
**2**	0	-232.33233	-0.857023956	0.8512606597
**3**	0	-232.2715	-0.887312838	0.8781443253
**4**	0	-232.27148	-0.887792945	0.8785650021
**5**	0	-232.27148	-0.887792945	0.8785650021

Model Information	
**Data Set**	WORK.UM
**Events Variable**	mortos
**Trials Variable**	total
**Number of Observations**	45
**Number of Events**	183
**Number of Trials**	450
**Name of Distribution**	Normal
**Log Likelihood**	-232.2714819

**Number of Observations Read**	45
**Number of Observations Used**	45
**Number of Events**	183
**Number of Trials**	450

Parameter Information	
**Parameter**	**Effect**
Intercept	Intercept
conc	conc

Last Evaluation of the Negative of the Gradient	
**Intercept**	**Log10(conc)**
7.2012394E-6	-8.345118E-6

Last Evaluation of the Negative of the Hessian		
	**Intercept**	**Log10(conc)**
**Intercept**	222.70488137	184.16708105
**Log10(conc)**	184.16708105	293.79921731

Algorithm converged.

Goodness-of-Fit Tests				
**Statistic**	**Value**	**DF**	**Value/DF**	**Pr > ChiSq**
**Pearson Chi-Square**	18.4046	43	0.4280	0.9996
**L.R. Chi-Square**	16.5095	43	0.3839	0.9999

Note: Since the Pearson Chi-Square is small (p > 0.1000), fiducial limits will be calculated using a z value of 1.96

toxicidade de fenitrotion para populacao `SL

The Probit Procedure

Response-Covariate Profile	
Response Levels	2
Number of Covariate Values	45

Type III Analysis of Effects			
Effect	DF	Wald Chi-Square	Pr > ChiSq
Log10(conc)	1	109.2214	<.0001

Analysis of Maximum Likelihood Parameter Estimates							
Parameter	DF	Estimate	Standard Error	95% Confidence Limits		Chi-Square	Pr > ChiSq
Intercept	1	-0.8878	0.0966	-1.0770	-0.6985	84.54	<.0001
Log10(conc)	1	0.8786	0.0841	0.7138	1.0433	109.22	<.0001
_C_	0	0.0000	0.0000	0.0000	0.0000		

Estimated Covariance Matrix		
	Intercept	Log10(conc)
Intercept	0.009323	-0.005844
Log10(conc)	-0.005844	0.007067

Probit Model in Terms of Tolerance Distribution	
MU	SIGMA
1.01050343	1.13821971

Estimated Covariance Matrix for Tolerance Parameters		
	MU	SIGMA
MU	0.006126	0.001913
SIGMA	0.001913	0.011862

toxicidade de fenitrotion para populacao `SL

The Probit Procedure

Probit Analysis on Log10(conc)			
Probability	Log10(conc)	95% Fiducial Limits	
0.01	-1.6374	-2.2298	-1.2246
0.02	-1.3271	-1.8511	-0.9601
0.03	-1.1303	-1.6113	-0.7919
0.04	-0.9822	-1.4313	-0.6649
0.05	-0.8617	-1.2851	-0.5615
0.06	-0.7592	-1.1608	-0.4732
0.07	-0.6693	-1.0521	-0.3956
0.08	-0.5888	-0.9549	-0.3259
0.09	-0.5156	-0.8666	-0.2624
0.10	-0.4482	-0.7855	-0.2038
0.15	-0.1692	-0.4518	0.0408
0.20	0.0526	-0.1897	0.2384
0.25	0.2428	0.0316	0.4114
0.30	0.4136	0.2265	0.5706
0.35	0.5719	0.4026	0.7227
0.40	0.7221	0.5648	0.8719
0.45	0.8675	0.7166	1.0213
0.50	1.0105	0.8609	1.1735
0.55	1.1535	1.0005	1.3303
0.60	1.2989	1.1384	1.4938
0.65	1.4491	1.2774	1.6661
0.70	1.6074	1.4210	1.8507
0.75	1.7782	1.5734	2.0524
0.80	1.9685	1.7410	2.2791
0.85	2.1902	1.9343	2.5455
0.90	2.4692	2.1754	2.8827
0.91	2.5366	2.2334	2.9644
0.92	2.6098	2.2963	3.0533
0.93	2.6903	2.3653	3.1511
0.94	2.7802	2.4424	3.2604
0.95	2.8827	2.5301	3.3852
0.96	3.0032	2.6329	3.5320
0.97	3.1513	2.7593	3.7127
0.98	3.3481	2.9269	3.9532
0.99	3.6584	3.1906	4.3326

toxicidade de fenitrotion para populacao `SL

The Probit Procedure

Probit Analysis on conc			
**Probability**	**conc**	**95% Fiducial Limits**	
**0.01**	0.02305	0.00589	0.05962
**0.02**	0.04709	0.01409	0.10961
**0.03**	0.07409	0.02447	0.16149
**0.04**	0.10419	0.03704	0.21630
**0.05**	0.13750	0.05187	0.27450
**0.06**	0.17411	0.06905	0.33638
**0.07**	0.21416	0.08870	0.40219
**0.08**	0.25776	0.11096	0.47215
**0.09**	0.30509	0.13596	0.54649
**0.10**	0.35630	0.16386	0.62544
**0.15**	0.67735	0.35337	1.09845
**0.20**	1.12864	0.64605	1.73133
**0.25**	1.74898	1.07550	2.57853
**0.30**	2.59191	1.68445	3.72090
**0.35**	3.73185	2.52678	5.28064
**0.40**	5.27398	3.67089	7.44500
**0.45**	7.37010	5.20664	10.50372
**0.50**	10.24480	7.25904	14.91110
**0.55**	14.24078	10.01247	21.39617
**0.60**	19.90069	13.75230	31.17214
**0.65**	28.12437	18.93991	46.36015
**0.70**	40.49360	26.36059	70.91147
**0.75**	60.00963	37.44701	112.81800
**0.80**	92.99365	55.08156	190.16181
**0.85**	154.95027	85.96289	351.12593
**0.90**	294.57147	149.76850	763.29248
**0.91**	344.01488	171.15779	921.29950
**0.92**	407.17731	197.82804	1130
**0.93**	490.09228	231.92121	1416
**0.94**	602.80573	276.91800	1821
**0.95**	763.32281	338.88836	2428
**0.96**	1007	429.48674	3404
**0.97**	1417	574.45208	5161
**0.98**	2229	845.05070	8978
**0.99**	4554	1551	21510

NOTE: The above quantiles and fiducial limits refer to effects due to the independent variable and do not include any effect due to the natural threshold.

toxicidade de fenitrotion para populacao `SL

The REG Procedure

Model: MODEL1

Dependent Variable: mort

**Number of Observations Read**	45
**Number of Observations Used**	45

Analysis of Variance					
**Source**	**DF**	**Sum of Squares**	**Mean Square**	**F Value**	**Pr > F**
**Model**	1	2.95227	2.95227	291.34	<.0001
**Error**	43	0.43573	0.01013		
**Corrected Total**	44	3.38800			

**Root MSE**	0.10066	**R-Square**	0.8714
**Dependent Mean**	0.40667	**Adj R-Sq**	0.8684
**Coeff Var**	24.75350		

Parameter Estimates					
**Variable**	**DF**	**Parameter Estimate**	**Standard Error**	**t Value**	**Pr > |t|**
**Intercept**	1	0.24570	0.01772	13.86	<.0001
**lconc**	1	0.25908	0.01518	17.07	<.0001
