# Supplementary material for: Lethal and behavioral effects of synthetic and organic insecticides on Spodoptera exigua and its predator Podisus maculiventris
Source: PLoS One. 2018 Nov 8;13(11):e0206789. doi: 10.1371/journal.pone.0206789 (PMC6224277; doi:10.1371/journal.pone.0206789)
Supplement: S8 File — (PDF) [file pone.0206789.s008.pdf]

toxicidade de fenitrotion para populacao `SL

Obs	conc	total	mortos	mort	lconc
1	0.5	9	1	0.11111	-0.30103
2	0.5	9	0	0.00000	-0.30103
3	0.5	9	1	0.11111	-0.30103
4	0.5	9	0	0.00000	-0.30103
5	1.0	9	1	0.11111	0.00000
6	1.0	9	1	0.11111	0.00000
7	1.0	9	2	0.22222	0.00000
8	1.0	9	1	0.11111	0.00000
9	2.5	9	3	0.33333	0.39794
10	2.5	9	3	0.33333	0.39794
11	2.5	9	3	0.33333	0.39794
12	2.5	9	3	0.33333	0.39794
13	5.0	9	4	0.44444	0.69897
14	5.0	9	4	0.44444	0.69897
15	5.0	9	4	0.44444	0.69897
16	5.0	9	4	0.44444	0.69897
17	10.0	9	5	0.55556	1.00000
18	10.0	9	5	0.55556	1.00000
19	10.0	9	5	0.55556	1.00000
20	10.0	9	6	0.66667	1.00000
21	25.0	9	7	0.77778	1.39794
22	25.0	9	7	0.77778	1.39794
23	25.0	9	6	0.66667	1.39794
24	25.0	9	7	0.77778	1.39794
25	50.0	9	8	0.88889	1.69897
26	50.0	9	9	1.00000	1.69897
27	50.0	9	9	1.00000	1.69897
28	50.0	9	9	1.00000	1.69897

toxicidade de fenitrotion para populacao `SL

The Probit Procedure

Iteration History for Parameter Estimates				
Iter	Ridge	Loglikelihood	Intercept	Log10(conc)
0	0	-174.67309	0	0
1	0	-123.25451	-0.86678793	1.1262463681
2	0	-121.11173	-1.101032707	1.4184935323
3	0	-121.09611	-1.12392051	1.4461736872
4	0	-121.09611	-1.124126507	1.4464180018
5	0	-121.09611	-1.124126507	1.4464180018

Model Information	
Data Set	WORK.UM
Events Variable	mortos
Trials Variable	total
Number of Observations	28
Number of Events	118
Number of Trials	252
Name of Distribution	Normal
Log Likelihood	-121.0961068

Number of Observations Read	28
Number of Observations Used	28
Number of Events	118
Number of Trials	252

Parameter Information	
Parameter	Effect
Intercept	Intercept
conc	conc

Last Evaluation of the Negative of the Gradient	
Intercept	Log10(conc)
2.6850512E-7	-5.427773E-7

Last Evaluation of the Negative of the Hessian		
	Intercept	Log10(conc)
Intercept	117.6028193	86.546825557
Log10(conc)	86.546825557	102.54925397

Algorithm converged.

Goodness-of-Fit Tests				
Statistic	Value	DF	Value/DF	Pr > ChiSq
Pearson Chi-Square	8.0428	26	0.3093	0.9997
L.R. Chi-Square	11.0788	26	0.4261	0.9953

Note: Since the Pearson Chi-Square is small (p > 0.1000), fiducial limits will be calculated using a z value of 1.96

toxicidade de fenitrotion para populacao `SL

The Probit Procedure

Response-Covariate Profile	
Response Levels	2
Number of Covariate Values	28

Type III Analysis of Effects			
Effect	DF	Wald Chi-Square	Pr > ChiSq
Log10(conc)	1	81.2943	<.0001

Analysis of Maximum Likelihood Parameter Estimates							
Parameter	DF	Estimate	Standard Error	95% Confidence Limits		Chi-Square	Pr > ChiSq
Intercept	1	-1.1241	0.1498	-1.4177	-0.8305	56.31	<.0001
Log10(conc)	1	1.4464	0.1604	1.1320	1.7608	81.29	<.0001
_C_	0	0.0000	0.0000	0.0000	0.0000		

Estimated Covariance Matrix		
	Intercept	Log10(conc)
Intercept	0.022441	-0.018939
Log10(conc)	-0.018939	0.025735

Probit Model in Terms of Tolerance Distribution	
MU	SIGMA
0.77717956	0.69136308

Estimated Covariance Matrix for Tolerance Parameters		
	MU	SIGMA
MU	0.004085	0.000351
SIGMA	0.000351	0.005880

toxicidade de fenitrotion para populacao `SL

The Probit Procedure

Probit Analysis on Log10(conc)			
**Probability**	**Log10(conc)**	**95% Fiducial Limits**	
**0.01**	-0.83117	-1.28867	-0.52912
**0.02**	-0.64271	-1.05068	-0.37148
**0.03**	-0.52313	-0.90008	-0.27108
**0.04**	-0.43318	-0.78704	-0.19529
**0.05**	-0.36001	-0.69528	-0.13345
**0.06**	-0.29773	-0.61734	-0.08066
**0.07**	-0.24313	-0.54915	-0.03422
**0.08**	-0.19424	-0.48821	0.00748
**0.09**	-0.14977	-0.43291	0.04552
**0.10**	-0.10884	-0.38212	0.08064
**0.15**	0.06063	-0.17318	0.22745
**0.20**	0.19531	-0.00927	0.34627
**0.25**	0.31086	0.12918	0.45038
**0.30**	0.41463	0.25118	0.54621
**0.35**	0.51078	0.36168	0.63755
**0.40**	0.60202	0.46377	0.72699
**0.45**	0.69030	0.55960	0.81648
**0.50**	0.77718	0.65087	0.90759
**0.55**	0.86406	0.73910	1.00172
**0.60**	0.95233	0.82586	1.10027
**0.65**	1.04358	0.91284	1.20483
**0.70**	1.13973	1.00204	1.31748
**0.75**	1.24350	1.09605	1.44130
**0.80**	1.35905	1.19864	1.58126
**0.85**	1.49373	1.31618	1.74645
**0.90**	1.66320	1.46191	1.95647
**0.91**	1.70413	1.49683	2.00747
**0.92**	1.74859	1.53468	2.06296
**0.93**	1.79749	1.57618	2.12409
**0.94**	1.85209	1.62242	2.19248
**0.95**	1.91437	1.67502	2.27061
**0.96**	1.98754	1.73667	2.36257
**0.97**	2.07749	1.81225	2.47581
**0.98**	2.19707	1.91243	2.62664
**0.99**	2.38553	2.06980	2.86490

toxicidade de fenitrotion para populacao `SL

The Probit Procedure

Probit Analysis on conc			
Probability	conc	95% Fiducial Limits	
0.01	0.14751	0.05144	0.29572
0.02	0.22766	0.08898	0.42513
0.03	0.29983	0.12587	0.53570
0.04	0.36882	0.16329	0.63783
0.05	0.43650	0.20171	0.73544
0.06	0.50381	0.24136	0.83051
0.07	0.57131	0.28239	0.92422
0.08	0.63939	0.32493	1.01737
0.09	0.70832	0.36905	1.11050
0.10	0.77833	0.41484	1.20405
0.15	1.14981	0.67115	1.68831
0.20	1.56788	0.97889	2.21957
0.25	2.04580	1.34641	2.82087
0.30	2.59794	1.78310	3.51731
0.35	3.24178	2.29975	4.34063
0.40	3.99968	2.90921	5.33326
0.45	4.90119	3.62746	6.55353
0.50	5.98659	4.47576	8.08323
0.55	7.31235	5.48407	10.03972
0.60	8.96054	6.69671	12.59720
0.65	11.05544	8.18161	16.02619
0.70	13.79529	10.04702	20.77206
0.75	17.51850	12.47519	27.62454
0.80	22.85838	15.79932	38.12963
0.85	31.16961	20.71013	55.77637
0.90	46.04654	28.96715	90.46253
0.91	50.59739	31.39288	101.73391
0.92	56.05239	34.25118	115.60170
0.93	62.73169	37.68619	133.07399
0.94	71.13652	41.92004	155.76919
0.95	82.10519	47.31779	186.47149
0.96	97.17158	54.53392	230.44397
0.97	119.53382	64.90014	299.09716
0.98	157.42211	81.73842	423.29460
0.99	242.95765	117.43486	732.64966

NOTE: The above quantiles and fiducial limits refer to effects due to the independent variable and do not include any effect due to the natural threshold.

toxicidade de fenitrotion para populacao `SL

The REG Procedure

Model: MODEL1

Dependent Variable: mort

**Number of Observations Read**	28
**Number of Observations Used**	28

Analysis of Variance					
**Source**	**DF**	**Sum of Squares**	**Mean Square**	**F Value**	**Pr > F**
**Model**	1	2.55040	2.55040	875.92	<.0001
**Error**	26	0.07570	0.00291		
**Corrected Total**	27	2.62610			

**Root MSE**	0.05396	**R-Square**	0.9712
**Dependent Mean**	0.46825	**Adj R-Sq**	0.9701
**Coeff Var**	11.52367		

Parameter Estimates					
**Variable**	**DF**	**Parameter Estimate**	**Standard Error**	**t Value**	**Pr > |t|**
**Intercept**	1	0.15420	0.01472	10.48	<.0001
**lconc**	1	0.44931	0.01518	29.60	<.0001
