# Supplementary material for: Lethal and behavioral effects of synthetic and organic insecticides on Spodoptera exigua and its predator Podisus maculiventris
Source: PLoS One. 2018 Nov 8;13(11):e0206789. doi: 10.1371/journal.pone.0206789 (PMC6224277; doi:10.1371/journal.pone.0206789)
Supplement: S9 File — (PDF) [file pone.0206789.s009.pdf]

toxicidade de fenitrotion para populacao `SL

Obs	conc	total	mortos	mort	lconc
1	5	10	1	0.1	0.69897
2	5	10	1	0.1	0.69897
3	5	10	2	0.2	0.69897
4	10	10	3	0.3	1.00000
5	10	10	3	0.3	1.00000
6	10	10	4	0.4	1.00000
7	25	10	4	0.4	1.39794
8	25	10	4	0.4	1.39794
9	25	10	4	0.4	1.39794
10	50	10	4	0.4	1.69897
11	50	10	5	0.5	1.69897
12	50	10	5	0.5	1.69897
13	100	10	5	0.5	2.00000
14	100	10	5	0.5	2.00000
15	100	10	6	0.6	2.00000
16	250	10	7	0.7	2.39794
17	250	10	7	0.7	2.39794
18	250	10	7	0.7	2.39794
19	500	10	9	0.9	2.69897
20	500	10	9	0.9	2.69897
21	500	10	9	0.9	2.69897

toxicidade de fenitrotion para populacao `SL

The Probit Procedure

Iteration History for Parameter Estimates				
**Iter**	**Ridge**	**Loglikelihood**	**Intercept**	**Log10(conc)**
**0**	0	-145.56091	0	0
**1**	0	-122.73542	-1.44539702	0.8437233686
**2**	0	-122.47216	-1.62245016	0.9481480685
**3**	0	-122.47206	-1.626000963	0.9502588366
**4**	0	-122.47206	-1.626000963	0.9502588366

Model Information	
**Data Set**	WORK.UM
**Events Variable**	mortos
**Trials Variable**	total
**Number of Observations**	21
**Number of Events**	104
**Number of Trials**	210
**Name of Distribution**	Normal
**Log Likelihood**	-122.4720591

**Number of Observations Read**	21
**Number of Observations Used**	21
**Number of Events**	104
**Number of Trials**	210

Parameter Information	
**Parameter**	**Effect**
Intercept	Intercept
conc	conc

Last Evaluation of the Negative of the Gradient	
**Intercept**	**Log10(conc)**
-2.763936E-6	-0.000044691

Last Evaluation of the Negative of the Hessian		
	**Intercept**	**Log10(conc)**
**Intercept**	116.06012623	196.90112612
**Log10(conc)**	196.90112612	379.82329661

Algorithm converged.

Goodness-of-Fit Tests				
**Statistic**	**Value**	**DF**	**Value/DF**	**Pr > ChiSq**
**Pearson Chi-Square**	5.0337	19	0.2649	0.9994
**L.R. Chi-Square**	5.1282	19	0.2699	0.9993

Note: Since the Pearson Chi-Square is small (p > 0.1000), fiducial limits will be calculated using a z value of 1.96

toxicidade de fenitrotion para populacao `SL

The Probit Procedure

Response-Covariate Profile	
Response Levels	2
Number of Covariate Values	21

Type III Analysis of Effects			
Effect	DF	Wald Chi-Square	Pr > ChiSq
Log10(conc)	1	41.3316	<.0001

Analysis of Maximum Likelihood Parameter Estimates							
Parameter	DF	Estimate	Standard Error	95% Confidence Limits		Chi-Square	Pr > ChiSq
Intercept	1	-1.6260	0.2674	-2.1501	-1.1019	36.98	<.0001
Log10(conc)	1	0.9503	0.1478	0.6606	1.2400	41.33	<.0001
_C_	0	0.0000	0.0000	0.0000	0.0000		

Estimated Covariance Matrix		
	Intercept	Log10(conc)
Intercept	0.071499	-0.037065
Log10(conc)	-0.037065	0.021847

Probit Model in Terms of Tolerance Distribution	
MU	SIGMA
1.71111375	1.05234486

Estimated Covariance Matrix for Tolerance Parameters		
	MU	SIGMA
MU	0.009547	0.000371
SIGMA	0.000371	0.026794

toxicidade de fenitrotion para populacao `SL

The Probit Procedure

Probit Analysis on Log10(conc)			
**Probability**	**Log10(conc)**	**95% Fiducial Limits**	
**0.01**	-0.7370	-1.8286	-0.1441
**0.02**	-0.4501	-1.4191	0.0789
**0.03**	-0.2681	-1.1597	0.2208
**0.04**	-0.1312	-0.9649	0.3279
**0.05**	-0.0198	-0.8066	0.4152
**0.06**	0.0750	-0.6721	0.4897
**0.07**	0.1581	-0.5544	0.5552
**0.08**	0.2325	-0.4491	0.6140
**0.09**	0.3002	-0.3535	0.6677
**0.10**	0.3625	-0.2656	0.7172
**0.15**	0.6204	0.0964	0.9240
**0.20**	0.8254	0.3810	1.0913
**0.25**	1.0013	0.6218	1.2383
**0.30**	1.1593	0.8340	1.3744
**0.35**	1.3056	1.0254	1.5057
**0.40**	1.4445	1.2005	1.6368
**0.45**	1.5789	1.3619	1.7717
**0.50**	1.7111	1.5115	1.9137
**0.55**	1.8434	1.6514	2.0654
**0.60**	1.9777	1.7844	2.2287
**0.65**	2.1166	1.9140	2.4053
**0.70**	2.2630	2.0441	2.5979
**0.75**	2.4209	2.1794	2.8109
**0.80**	2.5968	2.3258	3.0523
**0.85**	2.8018	2.4927	3.3374
**0.90**	3.0597	2.6991	3.6998
**0.91**	3.1221	2.7485	3.7877
**0.92**	3.1897	2.8021	3.8834
**0.93**	3.2642	2.8609	3.9887
**0.94**	3.3473	2.9263	4.1065
**0.95**	3.4421	3.0008	4.2411
**0.96**	3.5534	3.0880	4.3994
**0.97**	3.6904	3.1950	4.5943
**0.98**	3.8724	3.3369	4.8537
**0.99**	4.1592	3.5598	5.2634

toxicidade de fenitrotion para populacao `SL

The Probit Procedure

Probit Analysis on conc			
Probability	conc	95% Fiducial Limits	
0.01	0.18323	0.01484	0.71763
0.02	0.35470	0.03810	1.19919
0.03	0.53935	0.06923	1.66269
0.04	0.73924	0.10843	2.12752
0.05	0.95535	0.15609	2.60131
0.06	1.18838	0.21276	3.08823
0.07	1.43904	0.27903	3.59102
0.08	1.70802	0.35557	4.11176
0.09	1.99608	0.44313	4.65222
0.10	2.30398	0.54250	5.21395
0.15	4.17281	1.24844	8.39381
0.20	6.69018	2.40453	12.34040
0.25	10.03039	4.18642	17.31090
0.30	14.42991	6.82320	23.68281
0.35	20.21267	10.60265	32.04212
0.40	27.82949	15.86853	43.33343
0.45	37.92055	23.01126	59.11460
0.50	51.41783	32.47307	81.97661
0.55	69.71927	44.81308	116.24830
0.60	94.99971	60.87004	169.30246
0.65	130.79880	82.03458	254.26939
0.70	183.21623	110.69789	396.15566
0.75	263.57825	151.14880	646.93379
0.80	395.17506	211.72298	1128
0.85	633.57656	310.93314	2175
0.90	1147	500.14228	5009
0.91	1324	560.44915	6133
0.92	1548	634.02449	7645
0.93	1837	725.86607	9743
0.94	2225	843.92802	12780
0.95	2767	1002	17421
0.96	3576	1225	25084
0.97	4902	1567	39290
0.98	7454	2172	71407
0.99	14429	3629	183381

NOTE: The above quantiles and fiducial limits refer to effects due to the independent variable and do not include any effect due to the natural threshold.

toxicidade de fenitrotion para populacao `SL

The REG Procedure

Model: MODEL1

Dependent Variable: mort

**Number of Observations Read**	21
**Number of Observations Used**	21

Analysis of Variance					
**Source**	**DF**	**Sum of Squares**	**Mean Square**	**F Value**	**Pr > F**
**Model**	1	1.07350	1.07350	268.30	<.0001
**Error**	19	0.07602	0.00400		
**Corrected Total**	20	1.14952			

**Root MSE**	0.06325	**R-Square**	0.9339
**Dependent Mean**	0.49524	**Adj R-Sq**	0.9304
**Coeff Var**	12.77261		

Parameter Estimates					
**Variable**	**DF**	**Parameter Estimate**	**Standard Error**	**t Value**	**Pr > |t|**
**Intercept**	1	-0.07663	0.03754	-2.04	0.0554
**lconc**	1	0.33660	0.02055	16.38	<.0001
