# Supplementary material for: Lethal and behavioral effects of synthetic and organic insecticides on Spodoptera exigua and its predator Podisus maculiventris
Source: PLoS One. 2018 Nov 8;13(11):e0206789. doi: 10.1371/journal.pone.0206789 (PMC6224277; doi:10.1371/journal.pone.0206789)
Supplement: S10 File — (PDF) [file pone.0206789.s010.pdf]

toxicidade de fenitrotion para populacao `SL

Obs	conc	total	mortos	mort	lconc
1	1.0	9	1	0.11111	0.00000
2	1.0	9	0	0.00000	0.00000
3	1.0	9	0	0.00000	0.00000
4	1.0	9	0	0.00000	0.00000
5	2.5	9	1	0.11111	0.39794
6	2.5	9	1	0.11111	0.39794
7	2.5	9	0	0.00000	0.39794
8	2.5	9	0	0.00000	0.39794
9	5.0	9	1	0.11111	0.69897
10	5.0	9	1	0.11111	0.69897
11	5.0	9	2	0.22222	0.69897
12	5.0	9	1	0.11111	0.69897
13	10.0	9	2	0.22222	1.00000
14	10.0	9	2	0.22222	1.00000
15	10.0	9	2	0.22222	1.00000
16	10.0	9	2	0.22222	1.00000
17	25.0	9	3	0.33333	1.39794
18	25.0	9	3	0.33333	1.39794
19	25.0	9	4	0.44444	1.39794
20	25.0	9	4	0.44444	1.39794
21	50.0	9	5	0.55556	1.69897
22	50.0	9	4	0.44444	1.69897
23	50.0	9	5	0.55556	1.69897
24	50.0	9	4	0.44444	1.69897
25	100.0	9	6	0.66667	2.00000
26	100.0	9	6	0.66667	2.00000
27	100.0	9	6	0.66667	2.00000
28	100.0	9	7	0.77778	2.00000
29	250.0	9	8	0.88889	2.39794
30	250.0	9	8	0.88889	2.39794
31	250.0	9	7	0.77778	2.39794
32	250.0	9	8	0.88889	2.39794

toxicidade de fenitrotion para populacao `SL

The Probit Procedure

Iteration History for Parameter Estimates				
**Iter**	**Ridge**	**Loglikelihood**	**Intercept**	**Log10(conc)**
**0**	0	-199.62639	0	0
**1**	0	-136.12024	-1.442203282	0.9125002814
**2**	0	-131.92351	-1.942054536	1.2090234771
**3**	0	-131.83829	-2.026925575	1.2581256775
**4**	0	-131.83824	-2.029125753	1.2593868532
**5**	0	-131.83824	-2.029125753	1.2593868532

Model Information	
**Data Set**	WORK.UM
**Events Variable**	mortos
**Trials Variable**	total
**Number of Observations**	32
**Number of Events**	104
**Number of Trials**	288
**Name of Distribution**	Normal
**Log Likelihood**	-131.8382359

**Number of Observations Read**	32
**Number of Observations Used**	32
**Number of Events**	104
**Number of Trials**	288

Parameter Information	
**Parameter**	**Effect**
Intercept	Intercept
conc	conc

Last Evaluation of the Negative of the Gradient	
**Intercept**	**Log10(conc)**
0.0000352987	6.1997821E-6

Last Evaluation of the Negative of the Hessian		
	**Intercept**	**Log10(conc)**
**Intercept**	128.20942114	181.30139159
**Log10(conc)**	181.30139159	309.76162923

Algorithm converged.

Goodness-of-Fit Tests				
**Statistic**	**Value**	**DF**	**Value/DF**	**Pr > ChiSq**
**Pearson Chi-Square**	9.1589	30	0.3053	0.9999
**L.R. Chi-Square**	8.9446	30	0.2982	0.9999

Note: Since the Pearson Chi-Square is small (p > 0.1000), fiducial limits will be calculated using a z value of 1.96

toxicidade de fenitrotion para populacao `SL

The Probit Procedure

Response-Covariate Profile	
Response Levels	2
Number of Covariate Values	32

Type III Analysis of Effects			
Effect	DF	Wald Chi-Square	Pr > ChiSq
Log10(conc)	1	84.6679	<.0001

Analysis of Maximum Likelihood Parameter Estimates							
Parameter	DF	Estimate	Standard Error	95% Confidence Limits		Chi-Square	Pr > ChiSq
Intercept	1	-2.0291	0.2127	-2.4461	-1.6122	90.97	<.0001
Log10(conc)	1	1.2594	0.1369	0.9911	1.5276	84.67	<.0001
_C_	0	0.0000	0.0000	0.0000	0.0000		

Estimated Covariance Matrix		
	Intercept	Log10(conc)
Intercept	0.045259	-0.026490
Log10(conc)	-0.026490	0.018733

Probit Model in Terms of Tolerance Distribution	
MU	SIGMA
1.61120131	0.79403719

Estimated Covariance Matrix for Tolerance Parameters		
	MU	SIGMA
MU	0.005377	0.001848
SIGMA	0.001848	0.007447

toxicidade de fenitrotion para populacao `SL

The Probit Procedure

Probit Analysis on Log10(conc)			
**Probability**	**Log10(conc)**	**95% Fiducial Limits**	
**0.01**	-0.23601	-0.70857	0.07971
**0.02**	-0.01955	-0.43714	0.26176
**0.03**	0.11778	-0.26547	0.37781
**0.04**	0.22109	-0.13669	0.46547
**0.05**	0.30513	-0.03222	0.53706
**0.06**	0.37665	0.05647	0.59822
**0.07**	0.43937	0.13402	0.65206
**0.08**	0.49552	0.20327	0.70046
**0.09**	0.54659	0.26608	0.74465
**0.10**	0.59360	0.32372	0.78549
**0.15**	0.78823	0.56027	0.95671
**0.20**	0.94292	0.74491	1.09615
**0.25**	1.07563	0.89988	1.21921
**0.30**	1.19481	1.03544	1.33333
**0.35**	1.30524	1.15731	1.44282
**0.40**	1.41003	1.26916	1.55052
**0.45**	1.51142	1.37371	1.65839
**0.50**	1.61120	1.47318	1.76796
**0.55**	1.71098	1.56960	1.88058
**0.60**	1.81237	1.66491	1.99769
**0.65**	1.91716	1.76110	2.12104
**0.70**	2.02759	1.86047	2.25304
**0.75**	2.14677	1.96592	2.39726
**0.80**	2.27948	2.08171	2.55951
**0.85**	2.43417	2.21508	2.75022
**0.90**	2.62880	2.38117	2.99190
**0.91**	2.67581	2.42106	3.05049
**0.92**	2.72688	2.46433	3.11422
**0.93**	2.78303	2.51181	3.18438
**0.94**	2.84575	2.56475	3.26283
**0.95**	2.91728	2.62501	3.35242
**0.96**	3.00131	2.69568	3.45781
**0.97**	3.10462	2.78239	3.58755
**0.98**	3.24195	2.89740	3.76025
**0.99**	3.45841	3.07823	4.03291

toxicidade de fenitrotion para populacao `SL

The Probit Procedure

Probit Analysis on conc			
Probability	conc	95% Fiducial Limits	
0.01	0.58076	0.19563	1.20147
0.02	0.95598	0.36547	1.82711
0.03	1.31154	0.54266	2.38677
0.04	1.66376	0.72998	2.92058
0.05	2.01895	0.92850	3.44394
0.06	2.38042	1.13886	3.96481
0.07	2.75023	1.36151	4.48809
0.08	3.12984	1.59688	5.01715
0.09	3.52040	1.84534	5.55451
0.10	3.92285	2.10729	6.10220
0.15	6.14094	3.63304	9.05123
0.20	8.76845	5.55789	12.47808
0.25	11.90231	7.94110	16.56566
0.30	15.66058	10.85038	21.54396
0.35	20.19494	14.36527	27.72194
0.40	25.70599	18.58509	35.52361
0.45	32.46546	23.64327	45.53928
0.50	40.85087	29.72908	58.60791
0.55	51.40213	37.11945	75.95917
0.60	64.91848	46.22813	99.46888
0.65	82.63425	57.69019	132.14053
0.70	106.56015	72.52192	179.07520
0.75	140.20751	92.45307	249.61149
0.80	190.31800	120.70079	362.66659
0.85	271.74901	164.08928	562.62297
0.90	425.40336	240.52878	981.51569
0.91	474.03529	263.67203	1123
0.92	533.18803	291.29143	1301
0.93	606.78422	324.94672	1529
0.94	701.05061	367.06970	1832
0.95	826.56359	421.70809	2251
0.96	1003	496.22715	2870
0.97	1272	605.88142	3869
0.98	1746	789.59351	5758
0.99	2873	1197	10787

NOTE: The above quantiles and fiducial limits refer to effects due to the independent variable and do not include any effect due to the natural threshold.

toxicidade de fenitrotion para populacao `SL

The REG Procedure

Model: MODEL1

Dependent Variable: mort

**Number of Observations Read**	32
**Number of Observations Used**	32

Analysis of Variance					
**Source**	**DF**	**Sum of Squares**	**Mean Square**	**F Value**	**Pr > F**
**Model**	1	2.51130	2.51130	418.41	<.0001
**Error**	30	0.18006	0.00600		
**Corrected Total**	31	2.69136			

**Root MSE**	0.07747	**R-Square**	0.9331
**Dependent Mean**	0.36111	**Adj R-Sq**	0.9309
**Coeff Var**	21.45386		

Parameter Estimates					
**Variable**	**DF**	**Parameter Estimate**	**Standard Error**	**t Value**	**Pr > |t|**
**Intercept**	1	-0.07536	0.02535	-2.97	0.0058
**lconc**	1	0.36403	0.01780	20.46	<.0001
