# Supplementary material for: Lethal and behavioral effects of synthetic and organic insecticides on Spodoptera exigua and its predator Podisus maculiventris
Source: PLoS One. 2018 Nov 8;13(11):e0206789. doi: 10.1371/journal.pone.0206789 (PMC6224277; doi:10.1371/journal.pone.0206789)
Supplement: S11 File — (PDF) [file pone.0206789.s011.pdf]

toxicidade de fenitrotion para populacao `SL

Obs	conc	total	mortos	mort	lconc
1	25	10	0	0.0	1.39794
2	25	10	1	0.1	1.39794
3	25	10	0	0.0	1.39794
4	50	10	1	0.1	1.69897
5	50	10	1	0.1	1.69897
6	50	10	1	0.1	1.69897
7	100	10	3	0.3	2.00000
8	100	10	3	0.3	2.00000
9	100	10	3	0.3	2.00000
10	250	10	5	0.5	2.39794
11	250	10	5	0.5	2.39794
12	250	10	6	0.6	2.39794
13	500	10	8	0.8	2.69897
14	500	10	8	0.8	2.69897
15	500	10	9	0.9	2.69897

toxicidade de fenitrotion para populacao `SL

The Probit Procedure

Iteration History for Parameter Estimates				
**Iter**	**Ridge**	**Loglikelihood**	**Intercept**	**Log10(conc)**
**0**	0	-103.97208	0	0
**1**	0	-69.255018	-3.507295899	1.5481771957
**2**	0	-67.09699	-4.622451778	2.0218795605
**3**	0	-67.056395	-4.799812963	2.0960365423
**4**	0	-67.056372	-4.804086162	2.0978134499
**5**	0	-67.056372	-4.804086162	2.0978134499

Model Information	
**Data Set**	WORK.UM
**Events Variable**	mortos
**Trials Variable**	total
**Number of Observations**	15
**Number of Events**	54
**Number of Trials**	150
**Name of Distribution**	Normal
**Log Likelihood**	-67.05637227

**Number of Observations Read**	15
**Number of Observations Used**	15
**Number of Events**	54
**Number of Trials**	150

Parameter Information	
**Parameter**	**Effect**
Intercept	Intercept
conc	conc

Last Evaluation of the Negative of the Gradient	
**Intercept**	**Log10(conc)**
0.0000157229	0.0000232703

Last Evaluation of the Negative of the Hessian		
	**Intercept**	**Log10(conc)**
**Intercept**	65.515010597	142.36186535
**Log10(conc)**	142.36186535	320.0667123

Algorithm converged.

Goodness-of-Fit Tests				
**Statistic**	**Value**	**DF**	**Value/DF**	**Pr > ChiSq**
**Pearson Chi-Square**	3.6426	13	0.2802	0.9944
**L.R. Chi-Square**	3.7504	13	0.2885	0.9936

Note: Since the Pearson Chi-Square is small (p > 0.1000), fiducial limits will be calculated using a z value of 1.96

toxicidade de fenitrotion para populacao `SL

The Probit Procedure

Response-Covariate Profile	
Response Levels	2
Number of Covariate Values	15

Type III Analysis of Effects			
Effect	DF	Wald Chi-Square	Pr > ChiSq
Log10(conc)	1	47.1736	<.0001

Analysis of Maximum Likelihood Parameter Estimates							
Parameter	DF	Estimate	Standard Error	95% Confidence Limits		Chi-Square	Pr > ChiSq
Intercept	1	-4.8041	0.6751	-6.1273	-3.4809	50.64	<.0001
Log10(conc)	1	2.0978	0.3054	1.4992	2.6965	47.17	<.0001
_C_	0	0.0000	0.0000	0.0000	0.0000		

Estimated Covariance Matrix		
	Intercept	Log10(conc)
Intercept	0.455758	-0.202716
Log10(conc)	-0.202716	0.093290

Probit Model in Terms of Tolerance Distribution	
MU	SIGMA
2.2900445	0.47668681

Estimated Covariance Matrix for Tolerance Parameters		
	MU	SIGMA
MU	0.003759	0.001183
SIGMA	0.001183	0.004817

toxicidade de fenitrotion para populacao `SL

The Probit Procedure

Probit Analysis on Log10(conc)			
Probability	Log10(conc)	95% Fiducial Limits	
0.01	1.18111	0.76234	1.42401
0.02	1.31105	0.94104	1.52824
0.03	1.39350	1.05395	1.59484
0.04	1.45552	1.13857	1.64525
0.05	1.50596	1.20716	1.68650
0.06	1.54890	1.26535	1.72182
0.07	1.58655	1.31618	1.75295
0.08	1.62027	1.36154	1.78100
0.09	1.65092	1.40264	1.80665
0.10	1.67915	1.44033	1.83041
0.15	1.79599	1.59458	1.93056
0.20	1.88885	1.71435	2.01298
0.25	1.96852	1.81424	2.08656
0.30	2.04007	1.90097	2.15560
0.35	2.10637	1.97826	2.22266
0.40	2.16928	2.04851	2.28939
0.45	2.23014	2.11347	2.35695
0.50	2.29004	2.17462	2.42623
0.55	2.34995	2.23324	2.49803
0.60	2.41081	2.29060	2.57319
0.65	2.47372	2.34796	2.65281
0.70	2.54002	2.40671	2.73841
0.75	2.61156	2.46859	2.83230
0.80	2.69123	2.53611	2.93825
0.85	2.78410	2.61344	3.06311
0.90	2.90094	2.70926	3.22170
0.91	2.92916	2.73221	3.26020
0.92	2.95982	2.75707	3.30208
0.93	2.99353	2.78434	3.34822
0.94	3.03118	2.81471	3.39982
0.95	3.07412	2.84925	3.45878
0.96	3.12457	2.88972	3.52815
0.97	3.18659	2.93931	3.61359
0.98	3.26904	3.00503	3.72739
0.99	3.39898	3.10821	3.90714

toxicidade de fenitrotion para populacao `SL

The Probit Procedure

Probit Analysis on conc			
Probability	conc	95% Fiducial Limits	
0.01	15.17418	5.78552	26.54657
0.02	20.46678	8.73046	33.74751
0.03	24.74543	11.32259	39.34067
0.04	28.54405	13.75849	44.18292
0.05	32.06007	16.11256	48.58511
0.06	35.39195	18.42239	52.70054
0.07	38.59707	20.71008	56.61801
0.08	41.71242	22.98995	60.39451
0.09	44.76352	25.27193	64.06941
0.10	47.76896	27.56325	67.67144
0.15	62.51588	39.31725	85.22339
0.20	77.42028	51.80275	103.03458
0.25	93.00882	65.19943	122.05524
0.30	109.66542	79.61093	143.08822
0.35	127.75189	95.11815	166.97951
0.40	147.66490	111.81734	194.71144
0.45	169.88045	129.85950	227.48400
0.50	195.00444	149.49120	266.82825
0.55	223.84408	171.09728	314.79454
0.60	257.52045	195.25474	374.27662
0.65	297.66083	222.82060	449.58620
0.70	346.75227	255.09702	547.53632
0.75	408.85082	294.16743	679.67313
0.80	491.17273	343.64740	867.45306
0.85	608.27313	410.62322	1156
0.90	796.05529	511.98315	1666
0.91	849.50266	539.76963	1821
0.92	911.64044	571.57729	2005
0.93	985.22318	608.61428	2230
0.94	1074	652.69699	2511
0.95	1186	706.72716	2876
0.96	1332	775.73992	3374
0.97	1537	869.58697	4108
0.98	1858	1012	5338
0.99	2506	1283	8075

NOTE: The above quantiles and fiducial limits refer to effects due to the independent variable and do not include any effect due to the natural threshold.

toxicidade de fenitrotion para populacao `SL

The REG Procedure

Model: MODEL1

Dependent Variable: mort

**Number of Observations Read**	15
**Number of Observations Used**	15

Analysis of Variance					
**Source**	**DF**	**Sum of Squares**	**Mean Square**	**F Value**	**Pr > F**
**Model**	1	1.25027	1.25027	247.27	<.0001
**Error**	13	0.06573	0.00506		
**Corrected Total**	14	1.31600			

**Root MSE**	0.07111	**R-Square**	0.9501
**Dependent Mean**	0.36000	**Adj R-Sq**	0.9462
**Coeff Var**	19.75214		

Parameter Estimates					
**Variable**	**DF**	**Parameter Estimate**	**Standard Error**	**t Value**	**Pr > |t|**
**Intercept**	1	-0.89921	0.08216	-10.95	<.0001
**lconc**	1	0.61763	0.03928	15.72	<.0001
