# Supplementary material for: Lethal and behavioral effects of synthetic and organic insecticides on Spodoptera exigua and its predator Podisus maculiventris
Source: PLoS One. 2018 Nov 8;13(11):e0206789. doi: 10.1371/journal.pone.0206789 (PMC6224277; doi:10.1371/journal.pone.0206789)
Supplement: S12 File — (PDF) [file pone.0206789.s012.pdf]

toxicidade de fenitrotion para populacao `SL

Obs	conc	total	mortos	mort	lconc
1	100	10	2	0.2	2.00000
2	100	10	2	0.2	2.00000
3	100	10	2	0.2	2.00000
4	250	10	3	0.3	2.39794
5	250	10	3	0.3	2.39794
6	250	10	4	0.4	2.39794
7	500	10	6	0.6	2.69897
8	500	10	6	0.6	2.69897
9	500	10	5	0.5	2.69897

toxicidade de fenitrotion para populacao `SL

The Probit Procedure

Iteration History for Parameter Estimates				
Iter	Ridge	Loglikelihood	Intercept	Log10(conc)
0	0	-62.383246	0	0
1	0	-54.864993	-3.386102263	1.2900904
2	0	-54.791489	-3.778287087	1.4406350638
3	0	-54.791468	-3.785028906	1.4432254142
4	0	-54.791468	-3.785028906	1.4432254142

Model Information	
Data Set	WORK.UM
Events Variable	mortos
Trials Variable	total
Number of Observations	9
Number of Events	33
Number of Trials	90
Name of Distribution	Normal
Log Likelihood	-54.79146813

Number of Observations Read	9
Number of Observations Used	9
Number of Events	33
Number of Trials	90

Parameter Information	
Parameter	Effect
Intercept	Intercept
conc	conc

Last Evaluation of the Negative of the Gradient	
Intercept	Log10(conc)
8.116359E-6	0.0000163883

Last Evaluation of the Negative of the Hessian		
	Intercept	Log10(conc)
Intercept	51.532810259	123.58092505
Log10(conc)	123.58092505	300.35026151

Algorithm converged.

Goodness-of-Fit Tests				
Statistic	Value	DF	Value/DF	Pr > ChiSq
Pearson Chi-Square	0.8655	7	0.1236	0.9967
L.R. Chi-Square	0.8806	7	0.1258	0.9965

Note: Since the Pearson Chi-Square is small (p > 0.1000), fiducial limits will be calculated using a z value of 1.96

toxicidade de fenitrotion para populacao `SL

The Probit Procedure

Response-Covariate Profile	
Response Levels	2
Number of Covariate Values	9

Type III Analysis of Effects			
Effect	DF	Wald Chi-Square	Pr > ChiSq
Log10(conc)	1	8.3121	0.0039

Analysis of Maximum Likelihood Parameter Estimates							
Parameter	DF	Estimate	Standard Error	95% Confidence Limits		Chi-Square	Pr > ChiSq
Intercept	1	-3.7850	1.2085	-6.1537	-1.4164	9.81	0.0017
Log10(conc)	1	1.4432	0.5006	0.4621	2.4244	8.31	0.0039
_C_	0	0.0000	0.0000	0.0000	0.0000		

Estimated Covariance Matrix		
	Intercept	Log10(conc)
Intercept	1.460507	-0.600934
Log10(conc)	-0.600934	0.250587

Probit Model in Terms of Tolerance Distribution	
MU	SIGMA
2.62261797	0.69289245

Estimated Covariance Matrix for Tolerance Parameters		
	MU	SIGMA
MU	0.015381	0.018716
SIGMA	0.018716	0.057759

toxicidade de fenitrotion para populacao `SL

The Probit Procedure

Probit Analysis on Log10(conc)			
**Probability**	**Log10(conc)**	**95% Fiducial Limits**	
**0.01**	1.01071	-1.95393	1.59105
**0.02**	1.19959	-1.36694	1.70643
**0.03**	1.31943	-0.99503	1.78015
**0.04**	1.40958	-0.71563	1.83598
**0.05**	1.48291	-0.48867	1.88170
**0.06**	1.54533	-0.29575	1.92088
**0.07**	1.60005	-0.12685	1.95548
**0.08**	1.64905	0.02414	1.98670
**0.09**	1.69362	0.16122	2.01533
**0.10**	1.73464	0.28718	2.04192
**0.15**	1.90448	0.80518	2.15547
**0.20**	2.03946	1.20974	2.25286
**0.25**	2.15527	1.54568	2.34754
**0.30**	2.25926	1.82798	2.45195
**0.35**	2.35563	2.05566	2.58262
**0.40**	2.44708	2.22388	2.75444
**0.45**	2.53555	2.34265	2.96465
**0.50**	2.62262	2.43204	3.19904
**0.55**	2.70969	2.50663	3.44822
**0.60**	2.79816	2.57426	3.70957
**0.65**	2.88960	2.63927	3.98460
**0.70**	2.98597	2.70456	4.27766
**0.75**	3.08997	2.77272	4.59621
**0.80**	3.20577	2.84682	4.95273
**0.85**	3.34075	2.93166	5.36984
**0.90**	3.51060	3.03691	5.89615
**0.91**	3.55162	3.06215	6.02345
**0.92**	3.59618	3.08951	6.16180
**0.93**	3.64518	3.11952	6.31400
**0.94**	3.69991	3.15297	6.48405
**0.95**	3.76232	3.19104	6.67808
**0.96**	3.83566	3.23566	6.90614
**0.97**	3.92581	3.29039	7.18664
**0.98**	4.04565	3.36297	7.55969
**0.99**	4.23453	3.47704	8.14798

toxicidade de fenitrotion para populacao `SL

The Probit Procedure

Probit Analysis on conc			
Probability	conc	95% Fiducial Limits	
0.01	10.24965	0.01112	38.99912
0.02	15.83401	0.04296	50.86642
0.03	20.86557	0.10115	60.27735
0.04	25.67916	0.19247	68.54616
0.05	30.40264	0.32459	76.15517
0.06	35.10161	0.50611	83.34507
0.07	39.81562	0.74670	90.25739
0.08	44.57122	1.05715	96.98490
0.09	49.38771	1.44952	103.59356
0.10	54.28009	1.93721	110.13346
0.15	80.25666	6.38535	143.04422
0.20	109.51282	16.20831	179.00312
0.25	142.97796	35.12983	222.60992
0.30	181.66230	67.29451	283.10862
0.35	226.79440	113.67339	382.49023
0.40	279.94691	167.44785	568.11378
0.45	343.20070	220.11531	921.83496
0.50	419.38990	270.41940	1581
0.55	512.49280	321.09309	2807
0.60	628.29017	375.19945	5124
0.65	775.53895	435.78063	9652
0.70	968.21349	506.47689	18952
0.75	1230	592.54151	39465
0.80	1606	702.78579	89687
0.85	2192	854.40041	234334
0.90	3240	1089	787315
0.91	3561	1154	1055474
0.92	3946	1229	1451452
0.93	4418	1317	2060624
0.94	5011	1422	3048259
0.95	5785	1553	4765210
0.96	6849	1721	8056415
0.97	8430	1952	15368731
0.98	11108	2307	36281660
0.99	17160	2999	140599386

NOTE: The above quantiles and fiducial limits refer to effects due to the independent variable and do not include any effect due to the natural threshold.

toxicidade de fenitrotion para populacao `SL

The REG Procedure

Model: MODEL1

Dependent Variable: mort

**Number of Observations Read**	9
**Number of Observations Used**	9

Analysis of Variance					
**Source**	**DF**	**Sum of Squares**	**Mean Square**	**F Value**	**Pr > F**
**Model**	1	0.19536	0.19536	55.51	0.0001
**Error**	7	0.02464	0.00352		
**Corrected Total**	8	0.22000			

**Root MSE**	0.05933	**R-Square**	0.8880
**Dependent Mean**	0.36667	**Adj R-Sq**	0.8720
**Coeff Var**	16.17976		

Parameter Estimates					
**Variable**	**DF**	**Parameter Estimate**	**Standard Error**	**t Value**	**Pr > |t|**
**Intercept**	1	-0.85086	0.16461	-5.17	0.0013
**lconc**	1	0.51467	0.06908	7.45	0.0001
