# Supplementary material for: Lethal and behavioral effects of synthetic and organic insecticides on Spodoptera exigua and its predator Podisus maculiventris
Source: PLoS One. 2018 Nov 8;13(11):e0206789. doi: 10.1371/journal.pone.0206789 (PMC6224277; doi:10.1371/journal.pone.0206789)
Supplement: S13 File — (PDF) [file pone.0206789.s013.pdf]

toxicidade de fenitrotion para populacao `SL

Obs	conc	total	mortos	mort	lconc
1	1.0	10	1	0.1	0.00000
2	1.0	10	1	0.1	0.00000
3	1.0	10	1	0.1	0.00000
4	2.5	10	2	0.2	0.39794
5	2.5	10	1	0.1	0.39794
6	2.5	10	1	0.1	0.39794
7	5.0	10	2	0.2	0.69897
8	5.0	10	2	0.2	0.69897
9	5.0	10	2	0.2	0.69897
10	10.0	10	2	0.2	1.00000
11	10.0	10	2	0.2	1.00000
12	10.0	10	3	0.3	1.00000
13	25.0	10	5	0.5	1.39794
14	25.0	10	5	0.5	1.39794
15	25.0	10	5	0.5	1.39794
16	50.0	10	8	0.8	1.69897
17	50.0	10	8	0.8	1.69897
18	50.0	10	7	0.7	1.69897
19	100.0	10	10	1.0	2.00000
20	100.0	10	9	0.9	2.00000
21	100.0	10	9	0.9	2.00000

toxicidade de fenitrotion para populacao `SL

The Probit Procedure

Iteration History for Parameter Estimates				
**Iter**	**Ridge**	**Loglikelihood**	**Intercept**	**Log10(conc)**
**0**	0	-145.56091	0	0
**1**	0	-102.74225	-1.349159008	1.0921293262
**2**	0	-100.96854	-1.708836358	1.3659223271
**3**	0	-100.95612	-1.742666944	1.3906502124
**4**	0	-100.95612	-1.742946735	1.3908503065
**5**	0	-100.95612	-1.742946735	1.3908503065

Model Information	
**Data Set**	WORK.UM
**Events Variable**	mortos
**Trials Variable**	total
**Number of Observations**	21
**Number of Events**	86
**Number of Trials**	210
**Name of Distribution**	Normal
**Log Likelihood**	-100.9561227

**Number of Observations Read**	21
**Number of Observations Used**	21
**Number of Events**	86
**Number of Trials**	210

Parameter Information	
**Parameter**	**Effect**
Intercept	Intercept
conc	conc

Last Evaluation of the Negative of the Gradient	
**Intercept**	**Log10(conc)**
3.8565764E-7	-1.930964E-8

Last Evaluation of the Negative of the Hessian		
	**Intercept**	**Log10(conc)**
**Intercept**	98.371990536	110.42749565
**Log10(conc)**	110.42749565	157.76556592

Algorithm converged.

Goodness-of-Fit Tests				
**Statistic**	**Value**	**DF**	**Value/DF**	**Pr > ChiSq**
**Pearson Chi-Square**	9.3970	19	0.4946	0.9663
**L.R. Chi-Square**	10.3128	19	0.5428	0.9448

Note: Since the Pearson Chi-Square is small (p > 0.1000), fiducial limits will be calculated using a z value of 1.96

toxicidade de fenitrotion para populacao `SL

The Probit Procedure

Response-Covariate Profile	
Response Levels	2
Number of Covariate Values	21

Type III Analysis of Effects			
Effect	DF	Wald Chi-Square	Pr > ChiSq
Log10(conc)	1	65.3949	<.0001

Analysis of Maximum Likelihood Parameter Estimates							
Parameter	DF	Estimate	Standard Error	95% Confidence Limits		Chi-Square	Pr > ChiSq
Intercept	1	-1.7429	0.2178	-2.1698	-1.3160	64.03	<.0001
Log10(conc)	1	1.3909	0.1720	1.0538	1.7279	65.39	<.0001
_C_	0	0.0000	0.0000	0.0000	0.0000		

Estimated Covariance Matrix		
	Intercept	Log10(conc)
Intercept	0.047441	-0.033206
Log10(conc)	-0.033206	0.029581

Probit Model in Terms of Tolerance Distribution	
MU	SIGMA
1.25315192	0.71898464

Estimated Covariance Matrix for Tolerance Parameters		
	MU	SIGMA
MU	0.005516	0.001436
SIGMA	0.001436	0.007905

toxicidade de fenitrotion para populacao `SL

The Probit Procedure

Probit Analysis on Log10(conc)			
**Probability**	**Log10(conc)**	**95% Fiducial Limits**	
**0.01**	-0.41946	-0.93889	-0.09249
**0.02**	-0.22346	-0.68372	0.06880
**0.03**	-0.09911	-0.52234	0.17164
**0.04**	-0.00556	-0.40128	0.24934
**0.05**	0.07053	-0.30307	0.31281
**0.06**	0.13529	-0.21970	0.36706
**0.07**	0.19208	-0.14679	0.41481
**0.08**	0.24293	-0.08169	0.45775
**0.09**	0.28917	-0.02264	0.49696
**0.10**	0.33174	0.03156	0.53320
**0.15**	0.50797	0.25400	0.68523
**0.20**	0.64804	0.42770	0.80915
**0.25**	0.76820	0.57355	0.91863
**0.30**	0.87612	0.70113	1.02034
**0.35**	0.97611	0.81575	1.11820
**0.40**	1.07100	0.92074	1.21483
**0.45**	1.16280	1.01850	1.31213
**0.50**	1.25315	1.11104	1.41156
**0.55**	1.34350	1.20018	1.51440
**0.60**	1.43530	1.28769	1.62195
**0.65**	1.53019	1.37545	1.73581
**0.70**	1.63019	1.46557	1.85817
**0.75**	1.73810	1.56072	1.99231
**0.80**	1.85826	1.66473	2.14363
**0.85**	1.99833	1.78410	2.32188
**0.90**	2.17457	1.93228	2.54816
**0.91**	2.21713	1.96782	2.60307
**0.92**	2.26338	2.00634	2.66281
**0.93**	2.31422	2.04859	2.72860
**0.94**	2.37101	2.09567	2.80218
**0.95**	2.43578	2.14923	2.88623
**0.96**	2.51187	2.21202	2.98513
**0.97**	2.60541	2.28901	3.10691
**0.98**	2.72977	2.39107	3.26907
**0.99**	2.92576	2.55143	3.52516

toxicidade de fenitrotion para populacao `SL

The Probit Procedure

Probit Analysis on conc			
Probability	conc	95% Fiducial Limits	
0.01	0.38067	0.11511	0.80819
0.02	0.59778	0.20715	1.17164
0.03	0.79596	0.30037	1.48469
0.04	0.98727	0.39694	1.77559
0.05	1.17633	0.49766	2.05501
0.06	1.36551	0.60298	2.32840
0.07	1.55626	0.71320	2.59905
0.08	1.74955	0.82854	2.86912
0.09	1.94612	0.94921	3.14021
0.10	2.14653	1.07538	3.41354
0.15	3.22086	1.79475	4.84431
0.20	4.44671	2.67733	6.44390
0.25	5.86414	3.74581	8.29152
0.30	7.51824	5.02497	10.47947
0.35	9.46482	6.54261	13.12792
0.40	11.77604	8.33174	16.39942
0.45	14.54800	10.43526	20.51766
0.50	17.91232	12.91353	25.79647
0.55	22.05467	15.85552	32.68880
0.60	27.24612	19.39522	41.87469
0.65	33.89935	23.73851	54.42643
0.70	42.67641	29.21270	72.13830
0.75	54.71415	36.36780	98.24568
0.80	72.15471	46.20964	139.19592
0.85	99.61657	60.82750	209.83391
0.90	149.47474	85.56233	353.31556
0.91	164.86717	92.85833	400.93157
0.92	183.39048	101.46995	460.05604
0.93	206.16883	111.83801	535.30050
0.94	234.96883	124.64304	634.13478
0.95	272.75732	141.00514	769.53950
0.96	324.98874	162.93623	966.33913
0.97	403.10077	194.53927	1279
0.98	536.74231	246.07860	1858
0.99	842.86942	355.98551	3351

NOTE: The above quantiles and fiducial limits refer to effects due to the independent variable and do not include any effect due to the natural threshold.

toxicidade de fenitrotion para populacao `SL

The REG Procedure

Model: MODEL1

Dependent Variable: mort

**Number of Observations Read**	21
**Number of Observations Used**	21

Analysis of Variance					
**Source**	**DF**	**Sum of Squares**	**Mean Square**	**F Value**	**Pr > F**
**Model**	1	1.76238	1.76238	142.06	<.0001
**Error**	19	0.23571	0.01241		
**Corrected Total**	20	1.99810			

**Root MSE**	0.11138	**R-Square**	0.8820
**Dependent Mean**	0.40952	**Adj R-Sq**	0.8758
**Coeff Var**	27.19790		

Parameter Estimates					
**Variable**	**DF**	**Parameter Estimate**	**Standard Error**	**t Value**	**Pr > |t|**
**Intercept**	1	-0.03824	0.04474	-0.85	0.4034
**lconc**	1	0.43570	0.03656	11.92	<.0001
