# Supplementary material for: Lethal and behavioral effects of synthetic and organic insecticides on Spodoptera exigua and its predator Podisus maculiventris
Source: PLoS One. 2018 Nov 8;13(11):e0206789. doi: 10.1371/journal.pone.0206789 (PMC6224277; doi:10.1371/journal.pone.0206789)
Supplement: S14 File — (PDF) [file pone.0206789.s014.pdf]

toxicidade de fenitrotion para populacao `SL

Obs	conc	total	mortos	mort	lconc
1	10	9	0	0.00000	1.00000
2	10	9	1	0.11111	1.00000
3	10	9	1	0.11111	1.00000
4	10	9	0	0.00000	1.00000
5	25	9	1	0.11111	1.39794
6	25	9	1	0.11111	1.39794
7	25	9	2	0.22222	1.39794
8	25	9	1	0.11111	1.39794
9	50	9	4	0.44444	1.69897
10	50	9	3	0.33333	1.69897
11	50	9	4	0.44444	1.69897
12	50	9	3	0.33333	1.69897
13	100	9	6	0.66667	2.00000
14	100	9	6	0.66667	2.00000
15	100	9	6	0.66667	2.00000
16	100	9	5	0.55556	2.00000
17	250	9	9	1.00000	2.39794
18	250	9	8	0.88889	2.39794
19	250	9	8	0.88889	2.39794
20	250	9	8	0.88889	2.39794

toxicidade de fenitrotion para populacao `SL

The Probit Procedure

Iteration History for Parameter Estimates				
**Iter**	**Ridge**	**Loglikelihood**	**Intercept**	**Log10(conc)**
**0**	0	-124.76649	0	0
**1**	0	-83.14048	-2.947247493	1.6281707281
**2**	0	-80.652132	-3.903161575	2.1429307831
**3**	0	-80.610161	-4.048716154	2.2202103861
**4**	0	-80.610142	-4.051843799	2.2218628717
**5**	0	-80.610142	-4.051843799	2.2218628717

Model Information	
**Data Set**	WORK.UM
**Events Variable**	mortos
**Trials Variable**	total
**Number of Observations**	20
**Number of Events**	77
**Number of Trials**	180
**Name of Distribution**	Normal
**Log Likelihood**	-80.61014219

**Number of Observations Read**	20
**Number of Observations Used**	20
**Number of Events**	77
**Number of Trials**	180

Parameter Information	
**Parameter**	**Effect**
Intercept	Intercept
conc	conc

Last Evaluation of the Negative of the Gradient	
**Intercept**	**Log10(conc)**
7.5836108E-6	4.296453E-6

Last Evaluation of the Negative of the Hessian		
	**Intercept**	**Log10(conc)**
**Intercept**	78.791600628	139.30026011
**Log10(conc)**	139.30026011	258.50929983

Algorithm converged.

Goodness-of-Fit Tests				
**Statistic**	**Value**	**DF**	**Value/DF**	**Pr > ChiSq**
**Pearson Chi-Square**	6.7211	18	0.3734	0.9923
**L.R. Chi-Square**	7.0716	18	0.3929	0.9895

Note: Since the Pearson Chi-Square is small (p > 0.1000), fiducial limits will be calculated using a z value of 1.96

toxicidade de fenitrotion para populacao `SL

The Probit Procedure

Response-Covariate Profile	
Response Levels	2
Number of Covariate Values	20

Type III Analysis of Effects			
Effect	DF	Wald Chi-Square	Pr > ChiSq
Log10(conc)	1	60.3867	<.0001

Analysis of Maximum Likelihood Parameter Estimates							
Parameter	DF	Estimate	Standard Error	95% Confidence Limits		Chi-Square	Pr > ChiSq
Intercept	1	-4.0518	0.5179	-5.0669	-3.0368	61.21	<.0001
Log10(conc)	1	2.2219	0.2859	1.6615	2.7823	60.39	<.0001
_C_	0	0.0000	0.0000	0.0000	0.0000		

Estimated Covariance Matrix		
	Intercept	Log10(conc)
Intercept	0.268219	-0.144532
Log10(conc)	-0.144532	0.081751

Probit Model in Terms of Tolerance Distribution	
MU	SIGMA
1.8236246	0.45007278

Estimated Covariance Matrix for Tolerance Parameters		
	MU	SIGMA
MU	0.002622	0.000415
SIGMA	0.000415	0.003354

toxicidade de fenitrotion para populacao `SL

The Probit Procedure

Probit Analysis on Log10(conc)			
Probability	Log10(conc)	95% Fiducial Limits	
0.01	0.77660	0.42316	0.99534
0.02	0.89929	0.58475	1.09580
0.03	0.97713	0.68693	1.15988
0.04	1.03569	0.76357	1.20832
0.05	1.08332	0.82573	1.24789
0.06	1.12386	0.87850	1.28171
0.07	1.15941	0.92464	1.31150
0.08	1.19124	0.96584	1.33828
0.09	1.22019	1.00321	1.36274
0.10	1.24683	1.03751	1.38535
0.15	1.35715	1.17828	1.48021
0.20	1.44483	1.28826	1.55750
0.25	1.52006	1.38072	1.62571
0.30	1.58761	1.46175	1.68896
0.35	1.65020	1.53472	1.74969
0.40	1.70960	1.60174	1.80953
0.45	1.76707	1.66431	1.86971
0.50	1.82362	1.72362	1.93119
0.55	1.88018	1.78075	1.99486
0.60	1.93765	1.83677	2.06159
0.65	1.99705	1.89280	2.13242
0.70	2.05964	1.95015	2.20876
0.75	2.12719	2.01050	2.29270
0.80	2.20242	2.07623	2.38763
0.85	2.29010	2.15142	2.49971
0.90	2.40042	2.24447	2.64230
0.91	2.42706	2.26674	2.67694
0.92	2.45601	2.29087	2.71463
0.93	2.48784	2.31732	2.75616
0.94	2.52339	2.34678	2.80263
0.95	2.56393	2.38028	2.85573
0.96	2.61156	2.41951	2.91823
0.97	2.67012	2.46760	2.99522
0.98	2.74796	2.53130	3.09778
0.99	2.87065	2.63130	3.25983

toxicidade de fenitrotion para populacao `SL

The Probit Procedure

Probit Analysis on conc			
Probability	conc	95% Fiducial Limits	
0.01	5.97859	2.64945	9.89337
0.02	7.93027	3.84369	12.46808
0.03	9.48704	4.86327	14.45045
0.04	10.85647	5.80185	16.15531
0.05	12.11493	6.69470	17.69648
0.06	13.30036	7.55960	19.12993
0.07	14.43482	8.40697	20.48793
0.08	15.53246	9.24358	21.79114
0.09	16.60303	10.07411	23.05372
0.10	17.65359	10.90197	24.28593
0.15	22.75905	15.07578	30.21424
0.20	27.85055	19.42066	36.09940
0.25	33.11732	24.02791	42.23876
0.30	38.69067	28.95647	48.86096
0.35	44.68918	34.25450	56.19366
0.40	51.23892	39.97063	64.49554
0.45	58.48815	46.16456	74.08086
0.50	66.62306	52.91992	85.34803
0.55	75.88944	60.36022	98.82341
0.60	86.62619	68.67001	115.23586
0.65	99.32232	78.12674	135.65030
0.70	114.72102	89.15681	161.71984
0.75	134.02755	102.44654	196.20091
0.80	159.37324	119.18860	244.13313
0.85	195.02714	141.71708	316.01796
0.90	251.42941	175.57652	438.83284
0.91	267.33877	184.81721	475.26442
0.92	285.76502	195.37590	518.36308
0.93	307.49494	207.64622	570.37896
0.94	333.72280	222.21912	634.79264
0.95	366.37721	240.03714	717.34590
0.96	408.84694	262.73270	828.37967
0.97	467.86290	293.49266	989.05040
0.98	559.70745	339.85778	1253
0.99	742.42136	427.85348	1819

NOTE: The above quantiles and fiducial limits refer to effects due to the independent variable and do not include any effect due to the natural threshold.

toxicidade de fenitrotion para populacao `SL

The REG Procedure

Model: MODEL1

Dependent Variable: mort

**Number of Observations Read**	20
**Number of Observations Used**	20

Analysis of Variance					
**Source**	**DF**	**Sum of Squares**	**Mean Square**	**F Value**	**Pr > F**
**Model**	1	1.95489	1.95489	279.33	<.0001
**Error**	18	0.12597	0.00700		
**Corrected Total**	19	2.08086			

**Root MSE**	0.08366	**R-Square**	0.9395
**Dependent Mean**	0.42778	**Adj R-Sq**	0.9361
**Coeff Var**	19.55634		

Parameter Estimates					
**Variable**	**DF**	**Parameter Estimate**	**Standard Error**	**t Value**	**Pr > |t|**
**Intercept**	1	-0.67578	0.06863	-9.85	<.0001
**lconc**	1	0.64955	0.03886	16.71	<.0001
