# Supplementary material for: Lethal and behavioral effects of synthetic and organic insecticides on Spodoptera exigua and its predator Podisus maculiventris
Source: PLoS One. 2018 Nov 8;13(11):e0206789. doi: 10.1371/journal.pone.0206789 (PMC6224277; doi:10.1371/journal.pone.0206789)
Supplement: S15 File — (PDF) [file pone.0206789.s015.pdf]

toxicidade de fenitrotion para populacao `SL

Obs	conc	total	mortos	mort	lconc
1	5	10	1	0.1	0.69897
2	5	10	1	0.1	0.69897
3	5	10	1	0.1	0.69897
4	10	10	1	0.1	1.00000
5	10	10	2	0.2	1.00000
6	10	10	2	0.2	1.00000
7	25	10	4	0.4	1.39794
8	25	10	4	0.4	1.39794
9	25	10	4	0.4	1.39794
10	50	10	5	0.5	1.69897
11	50	10	5	0.5	1.69897
12	50	10	6	0.6	1.69897
13	100	10	6	0.6	2.00000
14	100	10	6	0.6	2.00000
15	100	10	6	0.6	2.00000
16	250	10	8	0.8	2.39794
17	250	10	8	0.8	2.39794
18	250	10	9	0.9	2.39794
19	500	10	10	1.0	2.69897
20	500	10	9	0.9	2.69897
21	500	10	9	0.9	2.69897

toxicidade de fenitrotion para populacao `SL

The Probit Procedure

Iteration History for Parameter Estimates				
Iter	Ridge	Loglikelihood	Intercept	Log10(conc)
0	0	-145.56091	0	0
1	0	-107.02187	-1.809285436	1.0789820196
2	0	-105.80764	-2.204031839	1.3172691211
3	0	-105.803	-2.230147528	1.3332006906
4	0	-105.803	-2.230262579	1.3332714998
5	0	-105.803	-2.230262579	1.3332714998

Model Information	
Data Set	WORK.UM
Events Variable	mortos
Trials Variable	total
Number of Observations	21
Number of Events	107
Number of Trials	210
Name of Distribution	Normal
Log Likelihood	-105.8030043

Number of Observations Read	21
Number of Observations Used	21
Number of Events	107
Number of Trials	210

Parameter Information	
Parameter	Effect
Intercept	Intercept
conc	conc

Last Evaluation of the Negative of the Gradient	
Intercept	Log10(conc)
-1.041252E-8	-6.783172E-8

Last Evaluation of the Negative of the Hessian		
	Intercept	Log10(conc)
Intercept	102.32422841	172.37146456
Log10(conc)	172.37146456	326.27093394

Algorithm converged.

Goodness-of-Fit Tests				
Statistic	Value	DF	Value/DF	Pr > ChiSq
Pearson Chi-Square	3.1724	19	0.1670	1.0000
L.R. Chi-Square	4.1147	19	0.2166	0.9999

Note: Since the Pearson Chi-Square is small (p > 0.1000), fiducial limits will be calculated using a z value of 1.96

toxicidade de fenitrotion para populacao `SL

The Probit Procedure

Response-Covariate Profile	
Response Levels	2
Number of Covariate Values	21

Type III Analysis of Effects			
Effect	DF	Wald Chi-Square	Pr > ChiSq
Log10(conc)	1	63.8173	<.0001

Analysis of Maximum Likelihood Parameter Estimates							
Parameter	DF	Estimate	Standard Error	95% Confidence Limits		Chi-Square	Pr > ChiSq
Intercept	1	-2.2303	0.2980	-2.8144	-1.6461	56.00	<.0001
Log10(conc)	1	1.3333	0.1669	1.0062	1.6604	63.82	<.0001
_C_	0	0.0000	0.0000	0.0000	0.0000		

Estimated Covariance Matrix		
	Intercept	Log10(conc)
Intercept	0.088817	-0.046923
Log10(conc)	-0.046923	0.027855

Probit Model in Terms of Tolerance Distribution	
MU	SIGMA
1.67277451	0.75003478

Estimated Covariance Matrix for Tolerance Parameters		
	MU	SIGMA
MU	0.005500	-0.000139
SIGMA	-0.000139	0.008815

toxicidade de fenitrotion para populacao `SL

The Probit Procedure

Probit Analysis on Log10(conc)			
Probability	Log10(conc)	95% Fiducial Limits	
0.01	-0.07207	-0.66705	0.29789
0.02	0.13239	-0.39908	0.46502
0.03	0.26211	-0.22945	0.57147
0.04	0.35970	-0.10212	0.65180
0.05	0.43908	0.00126	0.71734
0.06	0.50664	0.08909	0.77330
0.07	0.56588	0.16596	0.82250
0.08	0.61892	0.23465	0.86668
0.09	0.66716	0.29701	0.90698
0.10	0.71157	0.35430	0.94419
0.15	0.89541	0.59009	1.09965
0.20	1.04153	0.77528	1.22540
0.25	1.16688	0.93189	1.33556
0.30	1.27946	1.07005	1.43697
0.35	1.38377	1.19528	1.53373
0.40	1.48276	1.31093	1.62872
0.45	1.57852	1.41929	1.72417
0.50	1.67277	1.52208	1.82196
0.55	1.76702	1.62086	1.92375
0.60	1.86279	1.71725	2.03117
0.65	1.96178	1.81307	2.14600
0.70	2.06609	1.91055	2.27051
0.75	2.17867	2.01255	2.40808
0.80	2.30402	2.12319	2.56420
0.85	2.45014	2.24934	2.74900
0.90	2.63398	2.40513	2.98446
0.91	2.67839	2.44240	3.04168
0.92	2.72663	2.48276	3.10398
0.93	2.77967	2.52700	3.17262
0.94	2.83891	2.57626	3.24943
0.95	2.90647	2.63228	3.33720
0.96	2.98585	2.69788	3.44052
0.97	3.08344	2.77827	3.56779
0.98	3.21316	2.88478	3.73735
0.99	3.41762	3.05200	4.00524

toxicidade de fenitrotion para populacao `SL

The Probit Procedure

Probit Analysis on conc			
**Probability**	**conc**	**95% Fiducial Limits**	
**0.01**	0.84710	0.21526	1.98557
**0.02**	1.35641	0.39896	2.91759
**0.03**	1.82858	0.58958	3.72792
**0.04**	2.28928	0.79047	4.48538
**0.05**	2.74838	1.00292	5.21607
**0.06**	3.21100	1.22770	5.93329
**0.07**	3.68027	1.46541	6.64505
**0.08**	4.15836	1.71654	7.35669
**0.09**	4.64688	1.98157	8.07205
**0.10**	5.14714	2.26099	8.79412
**0.15**	7.85983	3.89122	12.57915
**0.20**	11.00346	5.96049	16.80367
**0.25**	14.68533	8.54858	21.65502
**0.30**	19.03075	11.75037	27.35056
**0.35**	24.19751	15.67751	34.17694
**0.40**	30.39173	20.46137	42.53286
**0.45**	37.88996	26.25978	52.98753
**0.50**	47.07329	33.27217	66.36787
**0.55**	58.48236	41.76976	83.89795
**0.60**	72.91110	52.14919	107.44130
**0.65**	91.57528	65.02379	139.95735
**0.70**	116.43757	81.38613	186.42756
**0.75**	150.89168	102.93153	255.90387
**0.80**	201.38156	132.79672	366.61035
**0.85**	281.92631	177.55701	561.05250
**0.90**	430.50952	254.17342	964.84232
**0.91**	476.85619	276.94892	1101
**0.92**	532.87709	303.92119	1271
**0.93**	602.10067	336.51423	1488
**0.94**	690.09485	376.93316	1776
**0.95**	806.25411	428.82027	2174
**0.96**	967.94339	498.74450	2758
**0.97**	1212	600.16736	3697
**0.98**	1634	766.97453	5462
**0.99**	2616	1127	10121

NOTE: The above quantiles and fiducial limits refer to effects due to the independent variable and do not include any effect due to the natural threshold.

toxicidade de fenitrotion para populacao `SL

The REG Procedure

Model: MODEL1

Dependent Variable: mort

**Number of Observations Read**	21
**Number of Observations Used**	21

Analysis of Variance					
**Source**	**DF**	**Sum of Squares**	**Mean Square**	**F Value**	**Pr > F**
**Model**	1	1.75562	1.75562	785.35	<.0001
**Error**	19	0.04247	0.00224		
**Corrected Total**	20	1.79810			

**Root MSE**	0.04728	**R-Square**	0.9764
**Dependent Mean**	0.50952	**Adj R-Sq**	0.9751
**Coeff Var**	9.27937		

Parameter Estimates					
**Variable**	**DF**	**Parameter Estimate**	**Standard Error**	**t Value**	**Pr > |t|**
**Intercept**	1	-0.22180	0.02806	-7.90	<.0001
**lconc**	1	0.43045	0.01536	28.02	<.0001
