# Supplementary material for: Lethal and behavioral effects of synthetic and organic insecticides on Spodoptera exigua and its predator Podisus maculiventris
Source: PLoS One. 2018 Nov 8;13(11):e0206789. doi: 10.1371/journal.pone.0206789 (PMC6224277; doi:10.1371/journal.pone.0206789)
Supplement: S16 File — (PDF) [file pone.0206789.s016.pdf]

toxicidade de fenitrotion para populacao `SL

Obs	conc	total	mortos	mort	lconc
1	2.5	13	2	0.15385	0.39794
2	2.5	13	3	0.23077	0.39794
3	2.5	13	2	0.15385	0.39794
4	5.0	13	6	0.46154	0.69897
5	5.0	13	5	0.38462	0.69897
6	5.0	13	6	0.46154	0.69897
7	10.0	13	7	0.53846	1.00000
8	10.0	13	7	0.53846	1.00000
9	10.0	13	8	0.61538	1.00000
10	25.0	13	9	0.69231	1.39794
11	25.0	13	9	0.69231	1.39794
12	25.0	13	9	0.69231	1.39794
13	50.0	13	11	0.84615	1.69897
14	50.0	13	11	0.84615	1.69897
15	50.0	13	11	0.84615	1.69897
16	100.0	13	13	1.00000	2.00000
17	100.0	13	13	1.00000	2.00000
18	100.0	13	12	0.92308	2.00000

toxicidade de fenitrotion para populacao `SL

The Probit Procedure

Iteration History for Parameter Estimates				
**Iter**	**Ridge**	**Loglikelihood**	**Intercept**	**Log10(conc)**
**0**	0	-162.19644	0	0
**1**	0	-120.48285	-1.110372466	1.1673342959
**2**	0	-118.80345	-1.346351746	1.4447138426
**3**	0	-118.79188	-1.366397735	1.4694751072
**4**	0	-118.79188	-1.366558913	1.4696774725
**5**	0	-118.79188	-1.366558913	1.4696774725

Model Information	
**Data Set**	WORK.UM
**Events Variable**	mortos
**Trials Variable**	total
**Number of Observations**	18
**Number of Events**	144
**Number of Trials**	234
**Name of Distribution**	Normal
**Log Likelihood**	-118.7918839

**Number of Observations Read**	18
**Number of Observations Used**	18
**Number of Events**	144
**Number of Trials**	234

Parameter Information	
**Parameter**	**Effect**
Intercept	Intercept
conc	conc

Last Evaluation of the Negative of the Gradient	
**Intercept**	**Log10(conc)**
-4.553011E-7	-8.813261E-7

Last Evaluation of the Negative of the Hessian		
	**Intercept**	**Log10(conc)**
**Intercept**	114.41760182	123.94223232
**Log10(conc)**	123.94223232	162.59809418

Algorithm converged.

Goodness-of-Fit Tests				
**Statistic**	**Value**	**DF**	**Value/DF**	**Pr > ChiSq**
**Pearson Chi-Square**	4.6334	16	0.2896	0.9973
**L.R. Chi-Square**	6.1049	16	0.3816	0.9869

Note: Since the Pearson Chi-Square is small (p > 0.1000), fiducial limits will be calculated using a z value of 1.96

toxicidade de fenitrotion para populacao `SL

The Probit Procedure

Response-Covariate Profile	
Response Levels	2
Number of Covariate Values	18

Type III Analysis of Effects			
Effect	DF	Wald Chi-Square	Pr > ChiSq
Log10(conc)	1	61.2095	<.0001

Analysis of Maximum Likelihood Parameter Estimates							
Parameter	DF	Estimate	Standard Error	95% Confidence Limits		Chi-Square	Pr > ChiSq
Intercept	1	-1.3666	0.2239	-1.8055	-0.9277	37.24	<.0001
Log10(conc)	1	1.4697	0.1879	1.1015	1.8379	61.21	<.0001
_C_	0	0.0000	0.0000	0.0000	0.0000		

Estimated Covariance Matrix		
	Intercept	Log10(conc)
Intercept	0.050147	-0.038225
Log10(conc)	-0.038225	0.035288

Probit Model in Terms of Tolerance Distribution	
MU	SIGMA
0.92983593	0.6804214

Estimated Covariance Matrix for Tolerance Parameters		
	MU	SIGMA
MU	0.004431	-0.001705
SIGMA	-0.001705	0.007564

toxicidade de fenitrotion para populacao `SL

The Probit Procedure

Probit Analysis on Log10(conc)			
Probability	Log10(conc)	95% Fiducial Limits	
0.01	-0.65306	-1.25097	-0.28769
0.02	-0.46758	-1.00549	-0.13736
0.03	-0.34990	-0.85000	-0.04173
0.04	-0.26137	-0.73319	0.03038
0.05	-0.18936	-0.63829	0.08915
0.06	-0.12807	-0.55762	0.13927
0.07	-0.07432	-0.48698	0.18330
0.08	-0.02620	-0.42380	0.22281
0.09	0.01756	-0.36641	0.25881
0.10	0.05784	-0.31365	0.29201
0.15	0.22462	-0.09604	0.43030
0.20	0.35718	0.07565	0.54147
0.25	0.47090	0.22163	0.63816
0.30	0.57302	0.35128	0.72643
0.35	0.66766	0.46976	0.80990
0.40	0.75745	0.58018	0.89110
0.45	0.84433	0.68459	0.97208
0.50	0.92984	0.78441	1.05472
0.55	1.01534	0.88074	1.14085
0.60	1.10222	0.97461	1.23237
0.65	1.19202	1.06728	1.33132
0.70	1.28665	1.16048	1.44006
0.75	1.38877	1.25674	1.56172
0.80	1.50249	1.35985	1.70128
0.85	1.63505	1.47619	1.86780
0.90	1.80183	1.61870	2.08120
0.91	1.84211	1.65266	2.13320
0.92	1.88588	1.68940	2.18984
0.93	1.93400	1.72963	2.25230
0.94	1.98774	1.77438	2.32223
0.95	2.04903	1.82521	2.40219
0.96	2.12104	1.88470	2.49636
0.97	2.20957	1.95754	2.61243
0.98	2.32725	2.05397	2.76713
0.99	2.51273	2.20524	3.01166

toxicidade de fenitrotion para populacao `SL

The Probit Procedure

Probit Analysis on conc			
Probability	conc	95% Fiducial Limits	
0.01	0.22230	0.05611	0.51560
0.02	0.34074	0.09874	0.72886
0.03	0.44679	0.14125	0.90839
0.04	0.54781	0.18485	1.07245
0.05	0.64661	0.22999	1.22785
0.06	0.74462	0.27693	1.37806
0.07	0.84271	0.32585	1.52512
0.08	0.94145	0.37688	1.67035
0.09	1.04126	0.43012	1.81471
0.10	1.14246	0.48568	1.95888
0.15	1.67735	0.80161	2.69337
0.20	2.27603	1.19027	3.47916
0.25	2.95732	1.66583	4.34671
0.30	3.74130	2.24535	5.32637
0.35	4.65217	2.94955	6.45504
0.40	5.72075	3.80345	7.78212
0.45	6.98768	4.83716	9.37736
0.50	8.50817	6.08711	11.34268
0.55	10.35950	7.59866	13.83076
0.60	12.65373	9.43209	17.07539
0.65	15.56024	11.67554	21.44483
0.70	19.34859	14.47044	27.54594
0.75	24.47785	18.06090	36.45227
0.80	31.80483	22.90100	50.26652
0.85	43.15662	29.93600	73.75650
0.90	63.36231	41.56217	120.55891
0.91	69.52073	44.94288	135.89334
0.92	76.89121	48.91025	154.82628
0.93	85.90051	53.65739	178.77110
0.94	97.21587	59.48106	210.00436
0.95	111.95140	66.86716	252.45695
0.96	132.14179	76.68335	313.59154
0.97	162.01982	90.68650	409.67045
0.98	212.44701	113.23218	584.96895
0.99	325.63629	160.41415	1027

NOTE: The above quantiles and fiducial limits refer to effects due to the independent variable and do not include any effect due to the natural threshold.

toxicidade de fenitrotion para populacao `SL

The REG Procedure

Model: MODEL1

Dependent Variable: mort

**Number of Observations Read**	18
**Number of Observations Used**	18

Analysis of Variance					
**Source**	**DF**	**Sum of Squares**	**Mean Square**	**F Value**	**Pr > F**
**Model**	1	1.21178	1.21178	454.48	<.0001
**Error**	16	0.04266	0.00267		
**Corrected Total**	17	1.25444			

**Root MSE**	0.05164	**R-Square**	0.9660
**Dependent Mean**	0.61538	**Adj R-Sq**	0.9639
**Coeff Var**	8.39092		

Parameter Estimates					
**Variable**	**DF**	**Parameter Estimate**	**Standard Error**	**t Value**	**Pr > |t|**
**Intercept**	1	0.05703	0.02888	1.97	0.0658
**lconc**	1	0.46570	0.02184	21.32	<.0001
